# Quantitative tRNA-sequencing uncovers metazoan tissue-specific tRNA regulation

**DOI:** 10.1038/s41467-020-17879-x

**Published:** 2020-08-14

**Authors:** Otis Pinkard, Sean McFarland, Thomas Sweet, Jeff Coller

**Affiliations:** 1 Department of Genetics and Genome Sciences, Case Western Reserve University, Cleveland, OH 44106 USA; 2 Tevard Biosciences, LabCentral, Cambridge, MA 02139 USA; 3 Department of Nutrition, Case Western Reserve University, Cleveland, OH 44106 USA; 4 Department of Molecular Biology & Genetics and Department of Biology, Johns Hopkins School of Medicine, Baltimore, MD 21205 USA

**Keywords:** Next-generation sequencing, tRNAs

## Abstract

Transfer RNAs (tRNA) are quintessential in deciphering the genetic code; disseminating nucleic acid triplets into correct amino acid identity. While this decoding function is clear, an emerging theme is that tRNA abundance and functionality can powerfully impact protein production rate, folding, activity, and messenger RNA stability. Importantly, however, the expression pattern of tRNAs is obliquely known. Here we present Quantitative Mature tRNA sequencing (QuantM-tRNA seq), a technique to monitor tRNA abundance and sequence variants secondary to RNA modifications. With QuantM-tRNA seq, we assess the tRNA transcriptome in mammalian tissues. We observe dramatic distinctions in isodecoder expression and known tRNA modifications between tissues. Remarkably, despite dramatic changes in tRNA isodecoder gene expression, the overall anticodon pool of each tRNA family is similar across tissues. These findings suggest that while anticodon pools appear to be buffered via an unknown mechanism, underlying transcriptomic and epitranscriptomic differences suggest a more complex tRNA regulatory landscape.

## Abstract (web summary)

The relative abundance of specific tRNA can impact protein production rate, folding, and messenger RNA stability. Here the authors describe QuantM-tRNA seq — a method to monitor tRNA abundance and sequence variants — and uncover distinctions in isodecoder expression between tissues that are independent of the anticodon pool of each tRNA family.

## Introduction

Translation of the genetic code is clearly critical to all life. While the ribosome is the cellular instrument that executes translation, it relies upon transfer RNA (tRNA) to properly decipher the genetic information contained within messenger RNA (mRNA)^1^. tRNAs parlay codon identity into amino acid identity. Each tRNA is charged with high precision and fidelity to one of 20 amino acids; the amino acid matches the tRNAs triplet code as determined by its anticodon loop. Reading of the genetic code takes place in the ribosome one amino acid at a time via base pairing between the mRNA codon and the tRNA anticodon; in this way, each amino acid is brought to the ribosome and polymerized into the growing polypeptide chain in precisely the order specified within the DNA-encoded gene.

This singular nature of tRNA in deciphering the genetic code necessitates that they are subject to a high degree of processing and quality control. tRNA transcripts are small; typically ~70–80 nucleotides in size. They are transcribed by RNA polymerase III (Pol III) in the nucleus and undergo an extensive maturation process before utilization^2,3^. Pol III promoter elements are internal to the tRNA body, constraining sequence variation. In addition, all tRNAs undergo exo/endonucleolytic trimming events and post-transcriptional nucleotide modifications. Some tRNAs are spliced, and all are post-transcriptionally 3′ end modified with the trinucleotide C-C-A. In the cytoplasm, tRNAs are charged with their appropriate amino acid by specific aminoacyl tRNA synthetases^4^. Mature tRNAs exhibit extensive secondary cloverleaf and tertiary L-shaped structure and a loop structure containing the codon-specific reverse-complement trinucleotide (anticodon). Together, processing, modification, and genomically encoded structure cooperate to stabilize tRNA and serve as recognition features for aminoacyl tRNA synthetases and translation factors^5,6^.

tRNAs are present in all known forms of life. In mammals, tRNA anticodons directly complement only 47 (mouse) or 48 (human) of the 61 sense codons^7^. The other codons in the genetic code are recognized by non-cognate tRNA interactions in accordance with Crick’s wobble rules^8,9^. For example, eight codons that end in cytosine (C) such as alanine 5′-GCC-3′ have no tRNA with a guanosine (G) in the wobble position of the anticodon that would decode the 3′ C. Instead, the 5′ adenosine, the wobble site of alanine tRNA 5′-AGC-3′, is converted to inosine (5′-IGC-3′) expanding its capacity to decode C, A, or uracil (U)-ending codons through non-traditional Watson–Crick base pairing. Thus RNA modifications within the anticodon loop expand the decoding potential of some tRNA families^8,10^.

Despite only 20 amino acids and 61 codons, mammals are hypothesized to have well over 400 discrete tRNA genes^7^. tRNA transcripts that share the same trinucleotide anticodon sequence but are encoded by many distinct genes are termed isodecoders. In less complex eukaryotes, such as yeast, these genes generate full-length, mature tRNAs of identical sequence. In mammals, however, isodecoders generally have sequence distinctions beyond the conserved anticodon^6^. Many of these differences occur in tRNA regions that are important for RNA Pol III transcription, raising the possibility isodecoders are transcribed differently^11^. In addition, subtle variations between isodecoders may alter their function in translation^12^. An important question in tRNA biology is whether mammalian isodecoders have distinct functions, are differentially expressed, or simply reflect genetic redundancy. The importance of this understanding is clear given the recent finding that tRNA levels dramatically impact mRNA translation and may influence mRNA decay rates^13^.

Importantly, beautiful work from many groups has suggested that tRNA levels are not static, but rather dynamic in nature in both normal and disease states. Gingold et al. showed that distinct tRNA pools associate with proliferative mammalian cell states compared to differentiation states^14^. Dittmar et al. clearly showed that tRNA expression differed across human tissues^15^. Moreover, dysregulation of tRNA expression has been identified in a wide array of human diseases^16^. Consistently, tRNA levels vary greatly across different cancer types, and Goodarzi et al. carefully showed this can favor translation of a pro-metastatic state^17–19^. Together, these data suggest upregulation of certain tRNA genes is associated with the pathogenesis of human malignancies. In addition, mutations in aminoacyl tRNA synthetases, enzymes-mediating tRNA processing events, and tRNA base modification enzymes are associated with clinical neurodegenerative, neurocognitive, and intellectual disabilities^3,20^. Since it is becoming clear that tRNA levels can influence gene expression, a detailed understanding of the tRNA transcriptome is essential.

Most previous work has relied on monitoring tRNA levels by tedious hybridization-based approaches including array and northern blotting techniques. Hybridization-based techniques can provide bulk quantitation for some tRNAs with the same anticodon; however, they are unable to distinguish certain anticodon groups and isodecoders differing by only one or a few bases^15,21,22^. Moreover, arrays and northern blots do not provide information about potential tRNA modifications, which are considered vital for their function.

This need for improved resolution provided the impetus to standardize high-throughput sequencing methodologies capable of discerning tRNA genes at the isodecoder level. Next-generation RNA sequencing has revolutionized modern molecular biology for most types of transcripts, except tRNAs. Historically, tRNAs are recalcitrant to high-throughput sequencing due to the aforementioned base modifications and extensive structures. Many base modifications disrupt Watson/Crick base pairing and the inherent stem-loop structures impede first-strand synthesis by reverse transcriptase (RT). To circumvent these issues, the few published methods employ clever and diverse library preparation strategies. DM-tRNA-seq was the first protocol published by Zheng et al. specifically for the purposes of sequencing tRNA^23^. This protocol utilizes a more processive RT and a purified prokaryotic demethylase, AlkB, to remove a series of methyl groups from tRNA that cause RT stalling, thus increasing the fraction of longer cDNA products. Gogakos et al. developed Hydro-tRNA-seq to increase the uniformity of coverage across a given tRNA transcript through a limited fragmentation of tRNA during library preparation to avoid modified bases^24^. This fragmentation allows for priming of shorter tRNA fragments and cDNA synthesis. Shigematsu et al. put forth YAMAT-seq, the most recent protocol, which utilizes a double-stranded adapter ligated to the 5′ and 3′ termini of mature tRNA that differs from the non-specific adapter ligation and template switching steps of Hydro-tRNA-seq and DM-tRNA-seq, respectively^25^. Despite these innovative strategies, significant limitations to the current state-of-the-art still exist. For example, DM-tRNA-seq relies on template switching using TGIRT and gel purification of tRNA, two steps with potential to introduce bias^26^. Hydro-tRNA-seq by design generates shorter reads which are difficult to map, and thus may be missing some information. YAMAT-seq is unable to quantify a large number of tRNAs due to the requirement for full-length cDNA, thus highly structured and modified tRNA that RT cannot fully traverse are selected against. A major limitation of each of these protocols is the lack of bias assessment and extensive cross-validation to evaluate the accuracy of each technique. Nonetheless, these technologies have greatly improved our understanding of tRNA biology and led to important and seminal discoveries. We posit, however, that a more robust and facile means to sequence tRNAs would accelerate this area of research.

Herein we present Quantitative Mature tRNA sequencing (QuantM-seq), a simple high-throughput tRNA sequencing protocol utilizing commercially available reagents. In HEK293 cells, we first showed that QuantM-seq is highly reproducible and representative of tRNA levels across a broad range of expression levels, thus providing a robust survey of the tRNA transcriptome. Using QuantM-seq, we surveyed tRNA transcriptomes from mice and reveal the expression landscape is dramatically different between tissue types. Fascinatingly, we see a strong CNS-specific expression pattern for unique isodecoders. Moreover, the nature of library production allows us to use sequence variant information as a means to approximate select tRNA modifications, and we observe tissue-specific nucleotide variants, highly suggestive of modifications, in particular within the CNS, that could possibly affect isodecoder function/stability. Intriguingly, when isodecoder levels are pooled bioinformatically, the anticodon pools between distinct tissues is similar in sharp contrast to the vast differences observed in isodecoder expression. These results suggest that strong anticodon buffering occurs, reducing the expression of some isodecoders as others increase in expression. The mechanism and consequence of this buffering is unclear. In total, we strongly feel that QuantM-tRNA-seq provides the community with a quick and relatively easy means to robustly monitor tRNA levels and begin to explore potential tRNA modifications. Technologies such as these should greatly accelerate our understanding of how tRNA influences normal and disease states.

## Results

### High-throughput sequencing of mature tRNA by QuantM-tRNA-seq

**Fig. 1:**
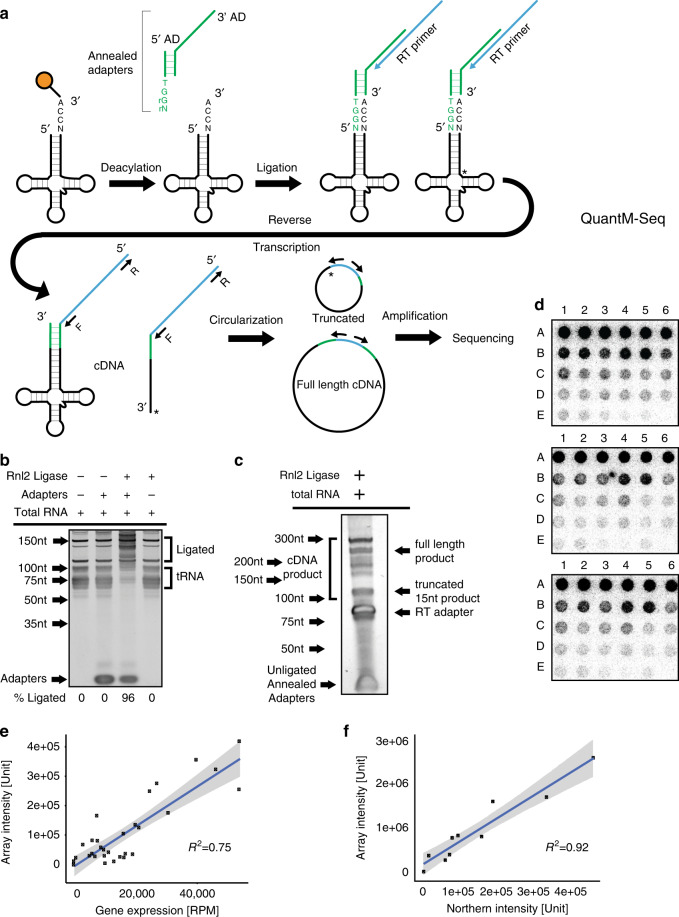
Quantitative mature tRNA sequencing (QuantM-seq). **a** Outline of QuantM-seq. tRNA depictions are in black, adapter depictions are in green, and sequences corresponding the RT primer are depicted in blue. The rG and rN at the end of the 5′ AD indicate ribonucleotides. **b** Polyacrylamide gel showing products and efficiency of adapter ligation onto tRNA. Rnl2: T4 RNA Ligase 2. Asterisk (*) indicates 5S and 5.8S ribosomal RNA bands. **c** Polyacrylamide gel showing products of reverse transcription (cDNA). Rnl2: T4 RNA Ligase 2. **d** Images of tRNA arrays; each array represents an independent replicate. For the probes spotted at each position see Source Data. **e** Scatter plot of reads per million derived from QuantM-seq versus array intensities derived from densitometry with a fitted linear trendline. Shaded area represents the 95% confidence interval of the linear trendline. **f** Scatter plot of northern blot versus array intensities derived from densitometry with a fitted linear trendline. Shaded area represents the 95% confidence interval of the linear trendline. Source data are provided as a Source Data file for (**b**–**f**).

We developed QuantM-tRNA-seq (Fig. 1a) to assay the relative abundance of mature tRNA. To test the assay, we utilized 1 µg of total RNA from HEK293 cells. Taking advantage of the 3′ terminal C-C-A added to functional tRNA, we optimized a splint ligation strategy to attach a complementary double-stranded adapter to the 5′ and 3′ termini (Fig. 1a)^27^. Double-stranded adapter ligation is highly efficient (96% ligation efficiency), specific for tRNA in the predicted range of 65–95 nucleotides, and dependent on both ligase and adapters (Fig. 1b). We then used SuperScript IV RT to generate cDNA due to a high level of processivity and thermostability. cDNA synthesized from ligated total RNA revealed a range of cDNA products; consistent with extensive tRNA base modification and structure that can inhibit RT. Notably, cDNA bands shorter than expected for full-length tRNA coincide with sites of highly modified bases known to inhibit RT, T-loop m^1^A, and the anticodon loop (Fig. 1c)^23^. In addition, a significant amount of full-length cDNA was obtained. This banding pattern was similar to DM-tRNA-seq with the shortest truncated cDNA bands coinciding with m^1^A56–59^23^. Following PAGE purification of all cDNA and subsequent ssDNA circularization, libraries were minimally amplified with seven cycles of PCR to add Illumina adapters and then subjected to high-throughput sequencing on an Illumina platform.

For data analysis, reads were first subjected to adapter and CCA trimming followed by alignment to the high-confidence set of human tRNA sequences annotated in gtRNAdb (Release 18; hg38) with Bowtie2. Under default conditions in Bowtie2 local mode, only reads that are 23 nucleotides or greater will map^28^. However, Fig. 1c shows that we have a significant proportion of ~15 nucleotide reads likely generated by stalling of reverse transcripase at m^1^A56–59 that would fail to map under default conditions^23^. To ensure all reads were able to be mapped, we set the minimum score threshold to allow for mapping of short reads that are ten nucleotides or greater.

Approximately 90% of reads mapped to gtRNAdb tRNAs, showing that the assay is very specific for tRNA. To select high-confidence reads, for further analysis we first plotted histograms of mapping quality (MAPQ) scores per read (Supplementary Fig. 1a, b). Reads were either MAPQ = 0, or ranged from MAPQ > 10 to MAPQ < 50, with increasing MAPQ indicating higher mapping confidence. In contrast with other RNA-seq protocols, tRNAs are short, relatively repetitive, and highly modified and thus prone to modification induced base misincorporation or truncation by RT^23,29,30^. Given these limitations inherent to tRNAs which would manifest as lower mapping quality relative to other types of RNA-seq, we selected a MAPQ of greater than 10 to calculate reads per million (RPM) per tRNA. Under the definition of MAPQ used by Bowtie2, this represents reads with >90% probability of the correct mapping. Reproducibility between biological replicate samples was excellent with Pearson’s *R*^2^ = 0.9999. Read length analysis revealed that while shorter reads are less likely to have a MAPQ > 10 (Supplementary Fig. 1c, d), there is considerable mappability of all read lengths. These data show that recovery of truncated tRNA cDNAs via circularization coupled with optimized mapping parameters allows us to include more tRNA reads. To complete these analyses, we counted reads corresponding to individual tRNA sequences and converted to RPM using established R packages (details in “Methods” and Supplementary Software)^31^.

Having shown that QuantM-seq is highly specific for tRNAs and reproducible, we next sought to extensively cross-validate the technique. Using total RNA isolated from HEK293 cells, we assessed abundance covering ~46% of known tRNA isodecoders (119 out of 256 unique tRNA sequences) by applying an orthogonal hybridization-based approach (tRNA arrays)^15^. Importantly, we utilized longer array probes antisense to full-length tRNAs (~70–80 nt; Supplementary Data 2). Longer probes allow limitations inherent to short probe hybridization approaches to be overcome^32–34^, and the original study describing tRNA arrays showed that these longer probes have comparable hybridization efficiencies^33^. To further ensure that probe efficiencies would be similar, we selected probes of similar length, GC content, melting temperature, and structure potential and showed that none of these potentially confounding characteristics correlated with probe signal from arrays (Supplementary Data 2).

We fixed 30 probes spanning the full-length of their cognate tRNA species to a nylon membrane in order from highest expressed by QuantM-seq to lowest expressed. As ligation of double-stranded adapters was specific for tRNA and highly efficient (96%; Fig. 1b), we ligated radiolabeled adapter to total RNA and hybridized to the tRNA probe array (Fig. 1d). tRNA abundance as assessed by QuantM-seq correlated strongly to the array signal (mean Pearson correlation coefficient across replicates *R*^2^ = 0.75; Fig. 1e). As a control, we further validated the array approach (ligation-dependent) by northern blot (ligation-independent) using 10 of the array probes spanning the range of array signal intensities. The northern signal for full-length tRNA and array intensities correlate strongly with a Pearson correlation coefficient of *R*^2^ = 0.92 (Fig. 1f), showing that array signal derives largely from full-length tRNA. Together, these results reveal that QuantM-seq provides comparable performance to hybridization approaches in assessing tRNA abundance.

### Comparison of QuantM-seq with established tRNA sequencing protocols

Several other groups have previously developed high-throughput tRNA-sequencing methodologies^23–25^. Each methodology utilizes different library preparation strategies with inherent biases and percentage of uniquely mapped reads. To compare QuantM-seq to previously published protocols, our data obtained from HEK293 cells were compared to publically available datasets from two previously published protocols, Hydro-seq and DM-tRNA-seq. In addition, we performed YAMAT-seq in parallel with QuantM-seq on the same HEK293 cell RNA. To control for potential differences in read processing, all datasets were subject to the same read quality control and alignment pipeline as QuantM-seq.

As a foundation for assay comparison, we first plotted the number of reads (depth of sequencing) from each dataset (Supplementary Fig. 1e). The percentage of reads assigned to a particular tRNA with a mapping quality score > 10 is an important metric detailing the efficiency of each protocol. This percentage varied greatly across protocols (Supplementary Fig. 1f). The YAMAT-seq protocol produced the highest percent of assigned reads at 87.4% of total reads. Hydro-seq had the lowest percentage of assigned mapped reads with a mean of 15.7% across three replicates of low coverage libraries. Gogakos et al. (Hydro-seq)^24^ conducted a second experiment dramatically increasing the number of reads (>100 M reads) for a single replicate, and this resulted in an increased percentage of assigned reads (Supplementary Fig. 1f; Hydro_HC). Interestingly, the percentage of assigned reads for libraries prepared by DM-tRNA-seq increased as the authors included more steps during library preparation. The percentage of assigned reads for libraries prepared from total RNA increased with demethylase treatment from 52.4% to 61.3%. Gel purification of tRNA prior to demethylase treatment and library preparation increased this percentage to 80.3%. However, it is important to note that DM-tRNA-seq assigned read percentages are likely inflated due to the fact that short reads less than 16 nucleotides were excluded from the Gene Expression Omnibus (GEO) record. Comparatively, 44.0% of QuantM-seq total reads were assigned to mature tRNA sequences.

**Fig. 2:**
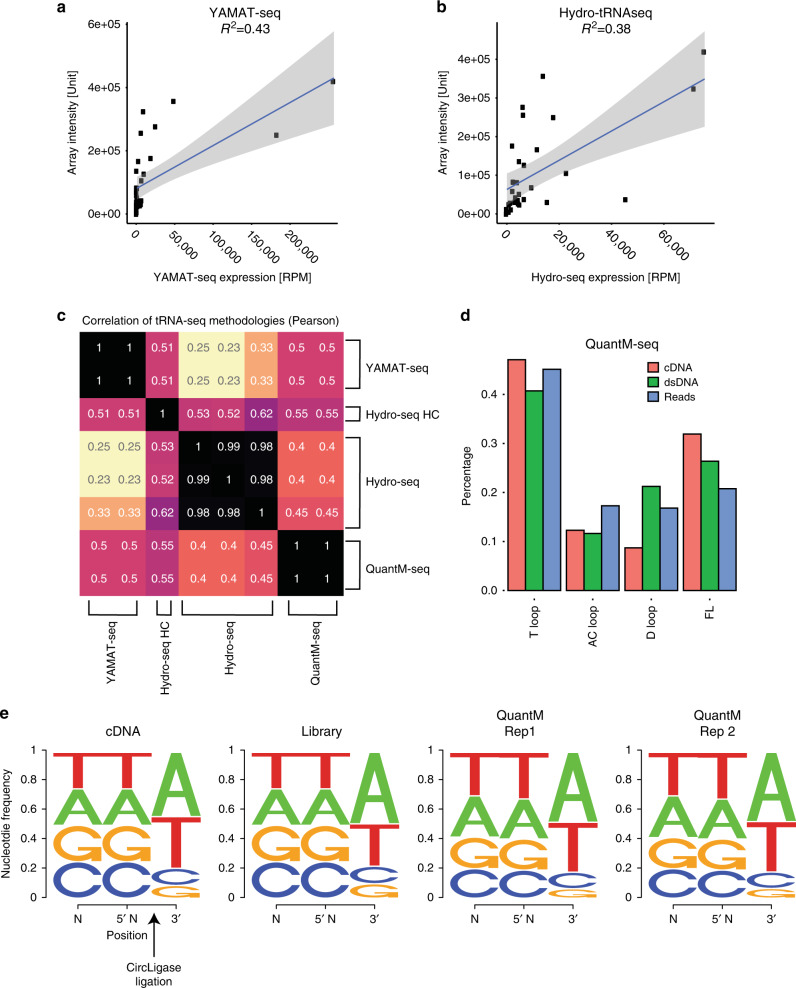
Comparison of QuantM-seq with other tRNA-seq protocols. **a** Scatter plot of reads per million derived from YAMAT-seq versus array intensities derived from densitometry with a fitted linear trendline. Shaded area represents the 95% confidence interval of the linear trendline. **b** Scatter plot of reads per million derived from Hydro-tRNA-seq versus array intensities derived from densitometry with a fitted linear trendline. Shaded area represents the 95% confidence interval of the linear trendline. **c** Pearson correlation coefficients between tRNA gene-level expression for each dataset. Hydro-seq HC denotes the high coverage library from ref. ^24^. **d** Bar chart depicting the average percentage of cDNA, dsDNA, or reads representing reverse transcriptase stalling or fall-off in the T-loop, anticodon (AC) loop, D-loop, or at the end of tRNA (full length; FL). Values were calculated from cDNA gel, Bioanalyzer trace, or reads respectively for *N* = 2 biological replicates in HEK293 cells. **e** Sequence logos showing the fraction of DNA bases near CircLigase ligated bases as inferred from cDNA gels (cDNA) or Bioanalyzer trace (Library), or calculated from reads (QuantM Rep 1 and QuantM Rep 2). See “Methods” for detailed calculations. Source data are provided as a Source Data file.

While QuantM-seq performs modestly with regards to read assignment to annotated tRNAs, the real question is how it performs relative to other techniques. Since YAMAT-seq was performed by us on the same RNA from HEK293 cells and Hydro-seq was performed on RNA from the same cells grown under the same conditions^24^, we compared these techniques directly to QuantM-seq. YAMAT-seq and Hydro-seq exhibited weaker correlations to tRNA arrays (*R*^2^ = 0.43 and 0.38, respectively; Fig. 2a, b) compared to QuantM-seq (*R*^2^ = 0.75; Fig. 1e). This is also reflected in weak correlation between these three techniques (Fig. 2c). Comparison of the distribution of mean RPM of 256 individual tRNA sequences revealed potential explanations for the disparity in expression values. Thirty of the 256 cytosolic tRNA genes detected reproducibly by QuantM-seq were not detected by YAMAT-seq (Supplementary Fig. 2a). In addition, YAMAT-seq showed higher variability between replicates and a general underrepresentation of most tRNA sequences relative to both QuantM-seq (Supplementary Fig. 2a) and tRNA arrays (Fig. 2a). Compared to QuantM-seq, Hydro-seq also showed higher variability between replicates (Supplementary Fig. 2c) and both over and underrepresented tRNAs relative to QuantM-seq (Supplementary Fig. 2b, c) and tRNA arrays (Fig. 2b).

### QuantM-seq exhibits minimal length and sequence bias

tRNA expression inferred from QuantM-seq cannot be directly compared to DM-tRNA-seq as this assay was performed on RNA isolated from HEK293T cells. However, we were able to assess length bias in both techniques going from cDNA to sequencing reads. For QuantM-seq, length of cDNA inferred from cDNA gels, dsDNA libraries by Bioanalyzer, and sequencing reads track closely, indicating that CircLigase and PCR are not introducing appreciable length bias (Fig. 2d). In contrast, DM-tRNA-seq reads from total RNA or purified tRNA exhibited significant skew toward longer reads or toward shorter reads respectively relative to cDNA (Supplementary Fig. 2e, f). Since read length is an important determinant of mappability, both kinds of skew are likely to contribute to inaccurate tRNA expression values. These skews are also likely underrepresented as the GEO record for this technique lacks raw reads <16 nt that would be generated by an RT stall in the T-loop. Short reads do have some mappability (Supplementary Fig. 1c, d), so loss of them also represents a loss of information. It is also important to note that gel purification of tRNA alone significantly alleviated m^1^A58 stalling of RT (Supplementary Fig. 2e, f). This raises the important question as to whether the poor recovery of highly structured, modified tRNA from PAGE gels is introducing bias. Together, these analyses reveal minimal length bias from cDNA to sequencing reads in QuantM-seq compared to DM-tRNA-seq.

While it was clear from DM-tRNA-seq that AlkB demethylase treatment could alleviate some stalling of RT at the T-loop^23^, the impact on quantitative power was less clear. To test if demethylase treatment could improve QuantM-seq, we treated HEK293 total RNA with a commercial demethylase preparation prior to performing QuantM-seq. Spike-in of 5 *Escherichia coli* tRNA to these libraries revealed QuantM-seq linearity over ~3.5 orders of magnitude from ~20 to 100,000 RPM (Supplementary Fig. 3a). Demethylase treatment resulted in a reduction in reads ending in the T-loop and an increase of reads that ended in the anticodon and D-loops, suggesting alleviation of stalling at methyl groups in the T-loop as seen by others^23^. However, similar to DM-tRNA-seq (Supplementary Fig. 2d), QuantM-seq expression values with or without demethylase treatment were highly correlated (Supplementary Fig. 3c). Further, demethylase treatment did not dramatically change correlation between QuantM-seq expression values and tRNA arrays (Supplementary Fig. 3d; *R*^2^ = 0.71), showing that demethylase treatment has minimal effects on our ability to quantitate tRNAs.

Lastly, we were able to predict an expected nucleotide frequency across the CircLigase ligation junction as we engineered two degenerate (N) bases with 25% representation of each base at the extreme 5′ end of the reverse transcription primer (Fig. 2e; left panel). We also knew expected nucleotide frequency for the majority of cDNA 3′ ends, as the shortest cDNA corresponds to RT stall at m^1^A^23^ and full-length cDNA end in T, and we know their relative proportion from cDNA gels (Fig. 2d). Comparing the predicted ligation junction sequence from cDNA to dsDNA library determined by Bioanalyzer length distributions to actual reads reveals minimal sequence bias introduced by cDNA purification, CircLigase ligation, PCR, and sequencing (Fig. 2e, compare all panels). We attempted to perform the same analysis for DM-tRNA-seq for the sake of comparison, however, the lack of short reads (<16 nt) in the GEO record prevented us from performing these calculations.

### tRNA anticodon pools are moderately regulated between tissues in mice

**Fig. 3:**
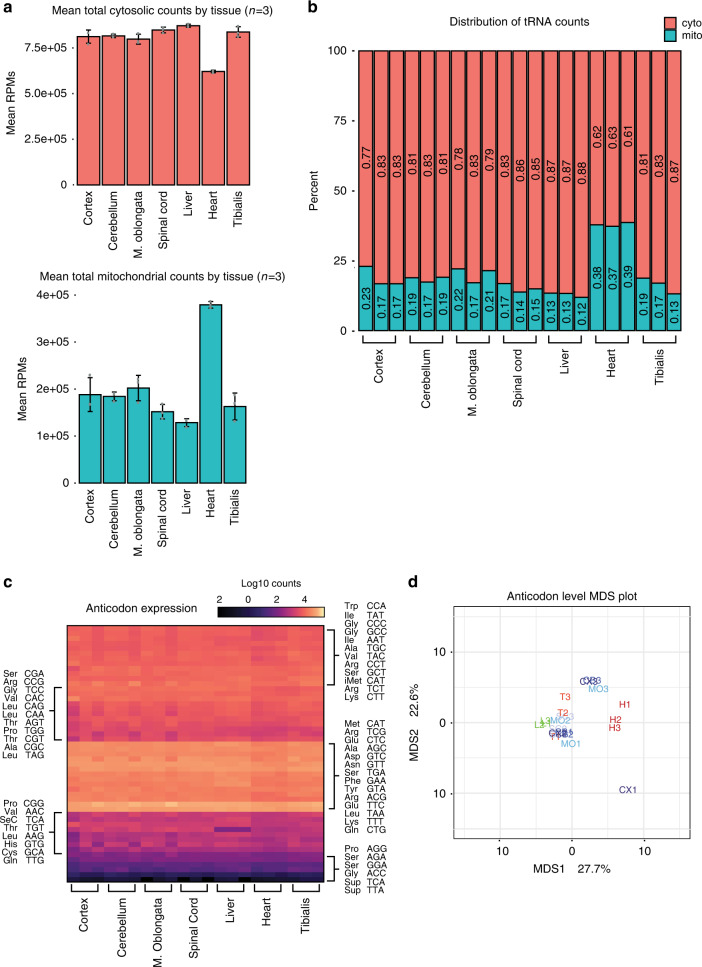
tRNA anticodon pools are similar across mouse tissues. **a** Bar chart with center showing the mean cytosolic tRNA reads per million (top) and mitochondrial tRNA reads per million (bottom) across seven mouse tissues. Error bars represent the standard deviation of *N* = 3 biological replicates. **b** Stacked bar charts showing the percentage of reads from each library that correspond to either cytoplasmic tRNA or mitochondrial tRNA. **c** tRNA reads were collapsed by known anticodon groups, log_10_ transformed, and then plotted as a heatmap. **d** Table used to generate (**c**) was used to create a multidimensional scaling plot to examine the distance between tissues with regards to tRNA expression at the anticodon level (Euclidean Distance). CNS tissues are labeled in shades of blue, non-CNS muscle tissues are in red, and the non-CNS liver is in green.

Having developed and extensively validated a sensitive high-throughput sequencing assay for tRNA expression, we set out to explore differences in mammalian tRNA expression. Previous studies outlined in Dittmar et al. and Gingold et al. using tRNA arrays suggested the presence of discrete expression profiles of tRNA across different tissues^14,15^. We obtained seven tissues from C57BL/6J wild-type mice in triplicate including four tissues derived from the central nervous system (cortex, cerebellum, medulla oblongata, and spinal cord) and three non-CNS tissues (heart, liver, and tibialis skeletal muscle). Total RNA was isolated from each tissue and tRNA libraries were generated using QuantM-seq (Fig. 1a). Following the same read processing pipeline as the HEK293 libraries, we aligned reads to high-confidence mouse tRNA genes annotated in gtRNAdb Release 18. The reproducibility of biological replicate samples was very high at an average of *r* = 0.97 or higher for each tissue (Supplementary Fig. 4b). A cursory analysis of the high-confidence reads revealed the contribution of cytosolic and mitochondrial tRNA genes to total reads differed significantly between heart and all other tissues. Surprisingly, reads mapped to cytosolic tRNA genes are relatively consistent across tissues with minor differences found only in heart (Fig. 3a). Interestingly, the contribution of mitochondrial tRNA genes to total gene expression is dramatically higher in the heart compared to all other tissues assayed (Fig. 3a, b).

As previous studies had assessed tRNA expression largely at the anticodon level, the 210 cytosolic tRNA isodecoders measured by QuantM-seq were summed by their respective anticodon sequence into the 47 genomically encoded anticodon classes. Differential anticodon class expression across tissues was analyzed using the established R package DEseq2 (details in “Methods” and Supplementary Software)^35^. Intriguingly, minor differences occurred between tissues at the level of anticodon expression with a significant decrease in Thr-TGT tRNAs in liver compared to all other tissues (11-fold-change and padj = 6.7E−22; Fig. 3c). Consistent with this, multidimensional scaling of the tRNA expression matrix showed high similarity between tissues (Fig. 3d). These findings suggest that tRNA anticodon pools are relatively stable across these seven tissues.

### tRNA isodecoders differ dramatically between tissues

**Fig. 4:**
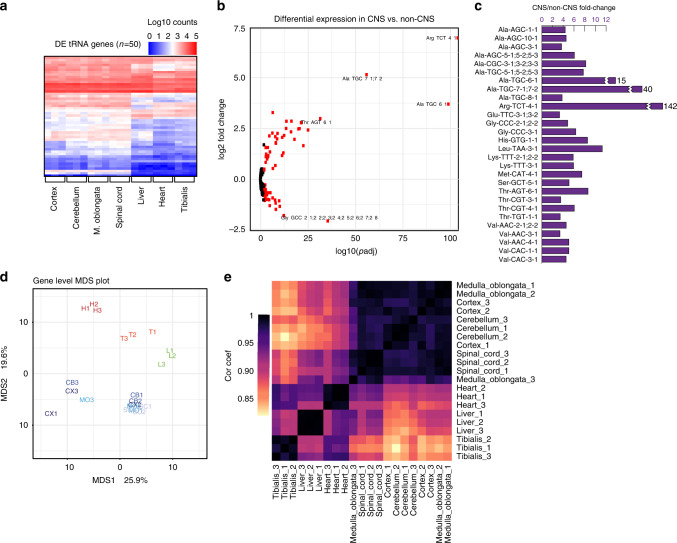
tRNA isodecoders change across mouse tissues. **a** Heatmap of log_10_-transformed tRNA isodecoder expression for isodecoders that significantly change across tissues (*p* < 0.01). Hierarchical clustering was used to group isodecoder expression along the *y*-axis. **b** The volcano plot of all isodecoders from the comparison of all samples lumped into two bins: CNS (cortex, cerebellum, medulla oblongata, spinal cord) versus non-CNS (liver, heart, tibialis). Isodecoders with a *p* value of 0.01 or less are colored red. Highly significant, tissue-specific isodecoders are labeled with their names. **c** Isodecoders that exhibit padj < 0.01 and CNS/non-CNS fold-change > 3 are depicted. **d** The expression matrix (RPM) used to generate (**a**) was used to generate a distance matrix and multidimensional scaling plot to examine the distance between tissues with regards to tRNA expression at the isodecoder level. CNS tissues are labeled in shades of blue, non-CNS muscle tissues are in red, and the non-CNS liver is in green. **e** Pearson correlation coefficients between tRNA isodecoder expression profiles of all tissues were plotted as a heatmap. Hierarchical clustering was used to group tissues along the *x* and *y* axes.

Given the similarity of tRNA expression profiles at the anticodon level, we next wanted to elucidate the potential regulation of individual tRNA isodecoders across tissues. We performed differential expression analysis of tRNA isodecoders across all tissues using DESeq2 (details in “Methods” and Supplementary Software)^35^. Of the 210 detected tRNAs, 41% (86 genes) of genes differed significantly in expression level between the seven tissues (padj < 0.01). Interestingly, a heatmap of differentially expressed isodecoders revealed CNS and non-CNS-associated tissues clustered with strikingly similar expression profiles across the four CNS-associated tissues (Fig. 4a). Differential expression analysis was performed following grouping of the four CNS tissues (cortex, cerebellum, medulla oblongata, and spinal cord) and three non-CNS tissues (heart, liver, tibialis). The expression levels of 57 genes differed significantly between CNS-associated tissues and non-CNS-associated tissues (27% of all isodecoders) (Fig. 4b). The most significant of these differentially expressed genes is a known CNS-specific isodecoder for Arginine, Arg-TCT-4-1, with 142-fold enrichment across CNS tissues compared to the three non-CNS tissues (*p* < 1E−200) (Fig. 4b, c). This isodecoder was identified previously as CNS-specific and having a CNS-specific function in translation^36^. In addition to identification of this previously reported CNS-specific tRNA isodecoder, we report a novel set of highly CNS-enriched Alanine TGC isodecoders including Ala-TGC-5-x, Ala-TGC-6-1, and Ala-TGC-7-x (Fig. 4b, c). Similar to Arg-TCT-4-1, all three genes were found to be greatly enriched at 8, 15, and 40-fold in the CNS-associated tissue group, respectively (*p* = 1.3E−27, 1.7E−115, 2.8E−87). The most significant isodecoder that is enriched fourfold in non-CNS tissue is Glycine GCC-2-x (*p* = 3.4E−48; Fig. 4b). In total, 28 isodecoders (13% of all isodecoders) exhibited a statistically significant (*p* < 0.01) greater than threefold enrichment in the CNS relative to non-CNS tissues (Fig. 4c).

Multidimensional scaling of isodecoder expression reveals the seven tissues in this study are dissimilar from one another. Most CNS tissues cluster together with potential outliers represented by samples isolated from one cortex and several CNS tissues from animal 3 (Fig. 4d). These could represent real individual differences or technical variability in tissue harvesting. Nevertheless, the distance matrix clearly illustrates the differences between CNS (blue) and non-CNS (green and red) tissues with each tissue co-localizing with other members of their respective CNS or non-CNS groups. Furthermore, correlation of all cytosolic genes revealed strong intercluster and weak intracluster correlation of CNS and non-CNS group members (Fig. 4e).

### Isodecoders underlying anticodon pools differ across tissues

**Fig. 5:**
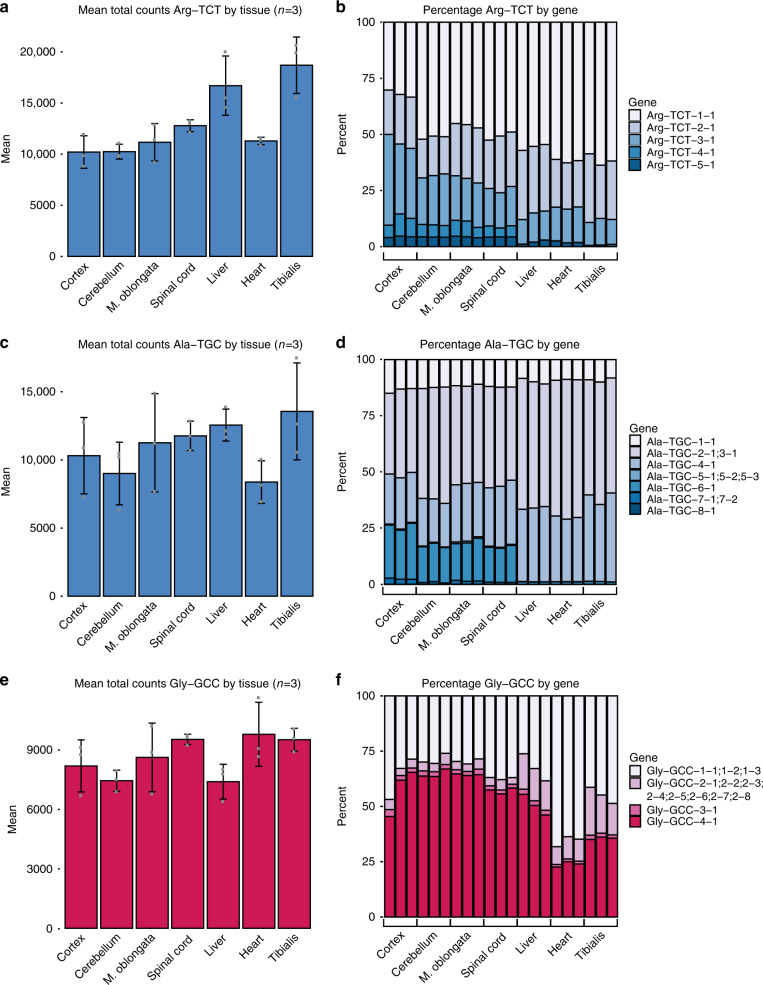
Isodecoder pools comprising anticodon pools differ across tissues. Bar charts with center showing mean total Arg-TCT (**a**), Ala-TGC (**c**), or Gly-GCC (**e**) reads per million across mouse tissues. Error bars represent the standard deviation of *N* = 3 biological replicates. Stacked bar charts for percentage of each isodecoder contributing to the pool of Arg-TCT (**b**), Ala-TGC (**d**), or Gly-GCC (**f**) across mouse tissues. Bar charts with blue shading represents examples of anticodon pools with CNS-enriched isodecoders. Red shading represents an example of an anticodon pool with non-CNS-enriched isodecoders.

Preceding analyses indicate that while anticodon pools are largely unchanged across tissues, the isodecoder pools that comprise anticodon pools differ significantly between CNS and non-CNS tissues. Next, we wanted to determine how individual tRNA isodecoders contribute to the considerably more stable anticodon expression levels across tissues. As mentioned previously, we first summed the RPM for each tRNA gene decoding a particular codon, and calculated an RPM per anticodon for each of the seven tissues (*n* = 3). From this, we calculated the percentage contribution of each tRNA gene for the 47 genomically encoded anticodon groups. Examples from anticodon classes with differentially expressed isodecoders revealed remarkable differences in percent contribution of constituent tRNA genes across tissues. The RPM of Arginine TCT tRNAs varied less than twofold across tissues (Fig. 5a). However, the previously defined CNS-specific isodecoder, Arg-TCT-4-1, contributed to 6% of total Arginine TCT RPM in the CNS, and ~0.03% in non-CNS tissues (Fig. 5b). Despite the relatively small contribution of Arg-TCT-4-1 to the Arginine TCT pool, mutation of this tRNA had dramatic effects on CNS-related phenotypes^36^, suggesting functional differences between isodecoders.

To determine the contribution of the newly identified CNS-specific isodecoders to the Alanine TGC anticodon class, we calculated the mean RPM by tissue (Fig. 5c) and found a marginal decrease (<2-fold) in heart relative to other tissues. We identified a similar pattern for the percentage of contribution to the Alanine TGC anticodon class for our newly identified set of three CNS-specific isodecoders. Ala-TGC-5-1, Ala-TGC-6-1, and Ala-TGC-7-1 contribute ~20% of mean RPM for the Alanine TGC anticodon across all CNS tissue classes (Fig. 5d). Interestingly, these three isodecoders contribute <2% of mean RPM in non-CNS associated tissues.

Similar to the relatively stable expression of Arg-TCT and Ala-TGC anticodons across tissues, the non-CNS tissue enriched Gly-GCC-2-x isodecoder did not result in dramatic tissue-specific changes in Glycine GCC anticodon expression (Fig. 5e). Interestingly, the average percentage contribution of the Gly-GCC-2-x isodecoder enriched in non-CNS associated tissues is 14% in non-CNS versus 4% in CNS tissues (Fig. 5f). Further analysis of heart versus other tissues revealed a heart-specific increase in Gly-GCC-1-x isodecoders contributing to a higher percentage of total RPM for the Glycine-GCC isoacceptor class (66% average in heart vs. 35% in all other tissues).

These findings indicate that while anticodon pools do not drastically change across tissues, the isodecoders that comprise these pools often do change. These results are highly suggestive of an unknown mechanism that buffers the overall amount of anticodons to offset tissue-specific changes in isodecoder expression.

### tRNA sequence variants differ across tissues

**Fig. 6:**
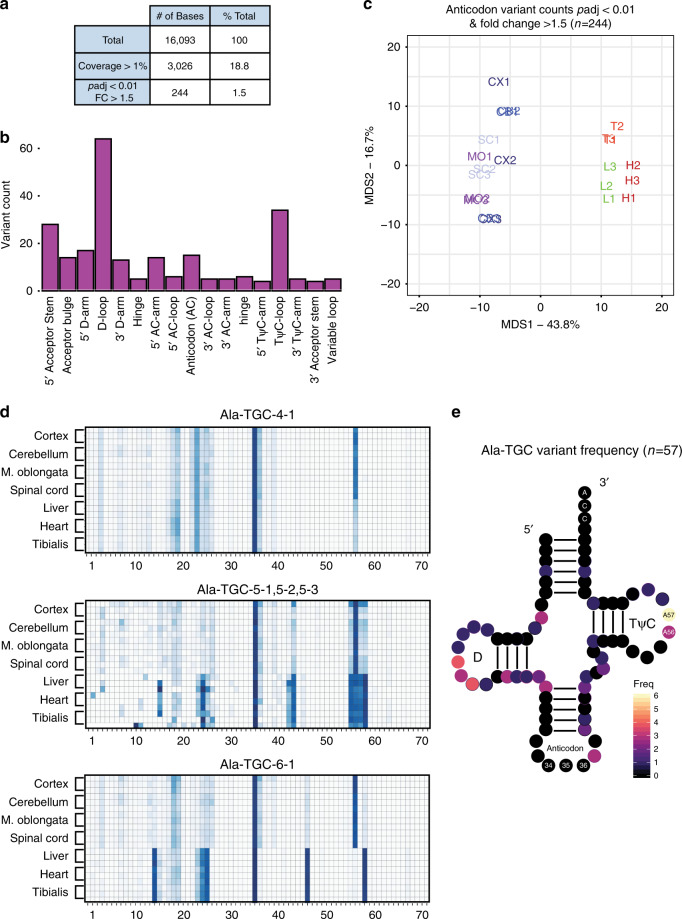
Variants in sequencing change across tissues in tRNA regions known to be modified. **a** Tabulation of QuantM-seq variant analyses. Total represents the total number of bases in gtRNAdb mouse tRNAs. Coverage > 1% represents the number of bases that have a variant fraction (variant count / read coverage) of at least 1% across all tissue samples. The last row represents bases that are at least 1% variant fraction, change with padj < 0.01 (Two-tailed, Benjamini–Hochberg), and exhibit an absolute fold-change of >1.5 across mouse tissues. **b** Histogram of significantly changed tRNA bases across seven tissues in mouse. All variant bases are binned by tRNA features. **c** Multidimensional scaling plot indicating distance between tissue samples with regards to differential variant fractions across tissues (Euclidean distance). CNS tissues are labeled in shades of blue, non-CNS muscle tissues are in red, and the non-CNS liver is in green. **d** Heatmaps for three isodecoders representing variant fractions at each tRNA position (*x*-axis) across each tissue sample (*y-*axis). The numbers below the plot indicate nucleotide position. **e** Two-dimensional representation of the Ala-TGC isoacceptor class showing counts of differential variants by position (*n* = 11 isodecoders).

tRNAs contain a high density of posttranscriptional base modification when compared to all other RNA classes with an average of 13 modifications per transcript^6^. Many of these modifications are absolutely essential for normal tRNA stability and function^37^. Of the over 100 identified base modifications found in tRNA, some are known to induce stalling and base misincorporation by RT, causing reproducible variation in cDNA products such as truncations and mutations^10,23,38^. Taking advantage of this relationship, we performed analyses to ask whether we can detect variants that indicate tRNA post-transcriptional base modification. We defined variants as the number of 5′ truncations (putative RT stalls) or mutations at a given base divided by the total read coverage at that base. Out of 16,093 distinct bases corresponding to 210 tRNA genes, we reliably detected 3026 bases that exhibited greater than 1% variation across all tissue samples, which indicates that 18.8% of the total bases detected by QuantM-seq represent sites of potential modification (Fig. 6a). This subset of variants generally exhibited intratissue reproducibility (Pearson’s *r*) greater than 0.85 (Supplementary Fig. 5a).

Next, we asked whether variant bases changed across tissues using DEXseq (details in “Methods”). We defined differential variants as bases whose adjusted *p* value was less than 0.01 and an absolute fold-change in variant frequency greater than 1.5. This revealed 244 (8%) potential modified bases which vary significantly in our analysis. Given the extensive secondary and tertiary structure required for normal function of mature tRNA, we set out to determine if these significant variant bases were enriched in particular structural regions. We found a great proportion of the significant variants to center around the TψC-loop, the anticodon loop, the entire D-arm, and the 5′ region of the acceptor stem (Fig. 6b). Consistent with these findings, modification sites are well documented in these four regions that mediate tRNA interactions with elongation factors, aminoacyl tRNA synthetases, ribosomal A, P, and E sites, as well as decoding of mRNA. Multidimensional scaling of the 244 significant variant frequencies revealed that tissues cluster into CNS and non-CNS tissues. This suggests differential regulation of variants between tissues (Fig. 6c).

Given this intriguing finding, we set out to better understand the relationship between differential tRNA isodecoder expression and differential variant frequencies between tissues by examining some examples. Within the Alanine TGC isoacceptor class, we identified multiple isodecoders that exhibit differential variant frequencies. Two of these isodecoders which are differentially expressed between tissues, Ala-TGC-5-x and Ala-TGC-6-1, also show multiple sites of differential variant frequencies between tissues (Fig. 6d), including at position 56, a likely site of m^1^A. Interestingly, an isodecoder which only modestly changes expression between tissues less than twofold, Ala-TGC-4-1, also exhibits differential variant frequencies comparable to the two isodecoders previously mentioned. This presents an unexpected finding suggesting significant variants are not exclusively found in differentially expressed tRNA genes.

To illustrate how this might impact the entire Ala-TGC anticodon pool, we present the distribution of significant variants for the 11 isodecoders in the Alanine TGC isoacceptor class as a consensus two-dimensional structure (Fig. 6e). Of note is the enrichment of significant adenosine variants at positions 56 and 57. Adenosine residues at positions 55–59 in the TψC-loop are methylated ubiquitously across all mature tRNA genes in all isoacceptor classes. The methylation at carbon-1 forms methyl-1-adenosine (m^1^A), which represents one of the most well characterized tRNA modifications with known regulatory functions^37^. Importantly, variants over A56 are shown to be m^1^A, as demethylase treatment of RNA from spinal cord causes a loss of CNS-specific variant signal (Supplementary Fig. 5b). Together, these analyses reveal that QuantM-seq can also be used to explore tRNA modifications across biological systems.

## Discussion

Historically, tRNAs have been difficult to sequence due to base modifications and extensive structures which impede first-strand synthesis by RT. To circumvent these issues, the few published methods employ clever and diverse library preparation strategies. However, each protocol has limitations. YAMAT-seq exhibits variability between technical replicates for some tRNAs and underrepresents many tRNAs. Hydro-seq exhibits improved coverage of the tRNA transcriptome compared to YAMAT-seq, but low correlation to tRNA arrays suggests that each of these two methods offer limited quantitative power (Fig. 2). DM-tRNA-seq utilizes TGIRT-mediated template switching, a step known to introduce bias^26^, exhibits length bias of sequencing reads relative to cDNA, and exhibits poor correlation between different implementations (Supplementary Fig. 2). Two iterations of DM-tRNA-seq implement tRNA purification from PAGE gels, a step that may add bias as well. Importantly, these techniques were not rigorously assessed for bias nor were the tRNA expression values derived from them cross-validated.

Here we present a high-throughput sequencing method, QuantM-tRNA-seq, for assessing the expression level of mature tRNA transcripts with isodecoder-level resolution. QuantM-tRNA-seq was subject to rigorous validation with orthogonal hybridization-based approaches accounting for 119 of the 256 measured tRNA genes in HEK293 cells offering high-confidence the data generated using this method accurately represents relative tRNA abundance in samples (Fig. 1). In addition to being extensively validated, this method exhibits minimal bias and improves upon previously published methodologies (Fig. 2). This was achieved with an efficient splint ligation strategy specific for mature tRNA transcripts containing a 3′ C-C-A, and a cDNA circularization strategy negating the need for transcript fragmentation or full-length cDNA synthesis by RT. Lastly, QuantM-seq was shown to have a wide dynamic range and not to require demethylase treatment of RNA for tRNA expression analysis (Supplementary Fig. 3). In summary, QuantM-Seq offers the best balance between coverage of the tRNA transcriptome, sequence depth, limited bias, cross-validation by tRNA arrays, ease of use, and reducing the need for RNA gel purification. Compared to other published methodologies, QuantM-tRNA-seq also greatly reduces the number of PCR amplification cycles necessary for reproducible library preparation with a comparable amount of input material. In the future, this may allow for reduced input tRNA-seq where sample material may be limiting.

To highlight the utility of QuantM-tRNA-seq, we assessed tRNA expression across seven mouse tissues at multiple levels: overall anticodon pools, tRNA isodecoder pools, and potential differences in nucleotide modification indirectly assessed by read variant analysis. Broadly speaking, anticodon pools changed modestly across tissues (Fig. 3) while isodecoders that comprise anticodon pools (Figs. 4 and 5) and nucleotide modifications (Fig. 6) exhibited tissue specificity. Consistent with our work here, Dittmar et al. detected differences in tRNA levels across human tissues^15^. However, a major limitation of these first generation tRNA arrays is the inability to distinguish isodecoders that differ by only a few bases and some anticodon pools. For example, these arrays could not distinguish Arg-CCG and Arg-TCG anticodons but instead sum them together as one signal. In addition, many isodecoders including the CNS-specific Arg-TCT and Ala-TGC we detected are unable to be distinguished by these probes.

Interestingly, the differences we detect reveal discrete signatures of isodecoder expression and nucleotide modification sites that are heavily CNS-enriched. Of particular note, QuantM-tRNA-seq revealed CNS enrichment of a tRNA gene, Arg-TCT-4-1, which was previously identified as CNS-specific, offering independent external validation of the protocol in two different isogenic mouse lines^36^. Mutated Arg-TCT-4-1 in C57/Black mice predispose these mice to neurodegenerative phenotypes, indicating functional importance despite this CNS-specific isodecoder comprising only 6% of total Arg-TCT tRNAs in the CNS (Fig. 5). Our analyses also revealed a novel set of 27 tRNA isodecoders enriched in CNS tissue representing several anticodon classes (Fig. 4). Further analysis of sequencing variants revealed that CNS-enriched Ala-TGC tRNAs also exhibit tissue-specific modification patterns. Specifically, each isodecoder exhibits significantly increased variation at A56 in the CNS (Fig. 6) that is alleviated by demethylase treatment (Supplementary Fig. 5). This base resides in the TψC-loop, is commonly modified with methyl groups (m^1^A), and is thought to influence tRNA stability and the ability to participate in translation^37^. It has been detected in other tRNA-seq as it causes significant RT stalling/mutation^23^. It is tempting to speculate that the increased expression of these isodecoders is linked to higher m^1^A56 methylation, possibly through increased tRNA stability, but more work needs to be done. To our knowledge this is the first known documentation of both novel CNS-specific isodecoders beyond Arg-TCT-4-1 as well as potential regulation of m^1^A56 modification across tissues.

Perhaps one of the most striking findings we observe herein is that while isodecoder expression can be quite distinct between tissue types, the overall decoding potential (based on summed anticodons) is relatively uniform. The observation of stable anticodon pools relative to differential isodecoder expression and modifications suggests two very interesting, non-mutually exclusive hypotheses. First, isodecoder sequence differences commonly occur in regions that RNA Pol III contacts for initiation. It is possible, therefore, that isodecoder genes are differentially transcribed in different tissues. In this scenario, the large number of isodecoder genes serves to buffer anticodon pools in a given tissue through reciprocal upregulation and downregulation of tRNA gene families at the transcriptional level. Second, it has been suggested that isodecoders may have different activities and that unique modifications influence stability and/or function^12,37^. Thus, while the level of anticodons between tissues appears uniform, the a priori assumption that the decoding potential is identical could be incorrect. The contribution of unique isodecoders to decoding potential may be distinct based on tissue-specific differences. These hypotheses warrant further investigation. With the ability to easily quantify tRNA at the anticodon, isodecoder, and variant levels, QuantM-tRNA-seq will be an essential tool for future studies aimed at testing these important ideas and probing tRNA biology in much more detail.

## Methods

### Cell culture and RNA isolation

HEK293 T-Rex Flp-IN cells (Thermo Fisher. cat# R78007) were cultured at 37 °C with 5% CO_2_ in complete Dulbecco’s modified essential media (Thermo Fisher) supplemented with 10% fetal bovine serum (Thermo Fisher) and 1% penicillin and streptomycin (Thermo Fisher). All passaging was performed with trypsin (Thermo Fisher) according to the manufacturer’s suggested conditions. Following passaging, cells were plated at 25% confluency in 10 cm tissue culture treated dishes and cultured for 48–72 h until ~90% confluent. At 90% confluency, media was aspirated and 1 mL of ice cold Trizol (Thermo Fisher) was added to the culture and mixed on ice for 30 s to assure a homogeneous solution. Samples were stored in Trizol at −80 °C until further processing. RNA was isolated according to manufacturer’s protocol with two 75% ethanol washes following isopropanol precipitation. Samples were resuspended in distilled H_2_O and stored at −80 °C until library preparation.

### Tissue sample preparation and RNA isolation

All mouse tissue samples were isolated from 31 to 37-day-old female C57B/6J mice using procedures approved by the PsychoGenics Institutional Animal Care and Use Committee (IACUC). Samples were received on dry ice, and stored as whole tissue at −80 °C. Samples were thawed on ice and 1 mL of Trizol was added per 100 mg of dissected whole tissue. On ice, samples were masticated and passed through successively higher gauges of needles to ensure a homogeneous mixture. Samples were stored at −80 °C until further processing. RNA was isolated according to the manufacturer’s suggested conditions with two 75% ethanol washes following isopropanol precipitation. Samples were resuspended in distilled H_2_O and stored at −80 °C until library preparation.

### Northern blotting

Total RNA (500 ng) was separated on a 7 M urea 6% denaturing polyacrylamide gel and transferred onto a Hybond-N + nylon transfer membrane (GE Life Sciences) then fixed by cross-linking in a UV Stratalinker 2400 using the auto-crosslink button twice. The blots were hybridized at 60 °C for 12 h in 2× SSC (1× SSC is 0.15 M NaCl and 0.015 M sodium citrate), 0.1% sodium dodecyl sulfate (SDS), and 10× Denhardt’s solution with ^32^P-end labeled probes specific for the full-length tRNA transcript (72–76 nucleotides; Supplementary Data 1 and 2). After two washes (each) in 2× SSC, 0.1% SDS for 20 min at room temperature and 0.5× SSC, 0.1% SDS for 60 min at 60 °C, the membrane was exposed to a storage phosphor screen for 15 min and analyzed on an Amersham Typhoon.

### tRNA array

100 nanograms of each probe (Supplementary Data 1 and 2) suspended in 0.5× TBE was aspirated onto a Hybond-N + nylon transfer membrane (GE Life Sciences) with a 96-well manifold under vacuum. The probes were UV cross-linked to the membrane as above. Arrays were stored at 4 °C in 0.5× TBE for future use. Radiolabeling of the double-stranded adapters was achieved by annealing 10 pmol of the 3′ adapter and 10 pmol of the 5′ adapter mix (2.5 pmol/µl each of the 5′-TGrGrA-3′, 5′-TGrGrT-3′, 5′-TGrGrG-3′, 5′-TGrGrC-3′ adapters) at 72 °C and reducing temperature to 37 °C by 0.1 °C /s. Annealed double-stranded adapters were ^32^P-end labeled by 5 U/µL of T4 polynucleotide kinase (NEB) under the manufacturer’s suggested conditions. The reaction was ethanol precipitated, ethanol washed, and resuspended in dH_2_O. The entire radiolabeled double-stranded adapter reaction was added to 300 ng of deacylated total RNA for ligation. The ligation reaction was carried out with 0.5 U/µL of T4 RNA ligase 2 (NEB) under manufacturer’s suggested conditions at 37 °C for 60 min then 4 °C for 60 min. The reaction was ethanol precipitated with glycoblue. 500,000 cpm of radiolabeled adapter-ligated total tRNA was hybridized to the cross-linked array membrane in 5 mL of hybridization buffer (2× SSC, 0.1% SDS, 10× Denhardt’s solution in dH_2_O) at 60 °C overnight. Membranes were washed for 5 min in 2× SSC, 0.1× SDS three times, then 0.5× SSC, 0.1× SDS for 60 min. All washes were performed at 60 °C. Membranes were exposed to a storage phosphor screen for 15 min and analyzed on an Amersham Typhoon.

### In vitro transcribed tRNA spike-in preparation

Five mature tRNA sequences derived from *E. coli* tRNA (gtRNAdb v8) were purchased as gBlocks (Integrated DNA Technologies; Supplementary Data 1) with a Hepatitis delta virus (HDV) ribozyme sequence at the 3′ end of the sequence to generate a precise CCA 3′ end. gBlocks were amplified using the Q5 2× master mix (NEB) and the T7 F and HDV R primers in Supplementary Data 1 according to the manufacturer’s suggested conditions. Design of the transcripts was in accordance with previously published protocols^39^. Amplification products of the appropriate length were purified from native agarose gels stained with 0.05% EtBr using the QiaQuick gel extraction kit (Qiagen). Up to 1 µg of double-stranded template DNA was added to the HiScribe T7 High Yield RNA synthesis kit (NEB) and transcribed according to the manufacturer’s suggested conditions. The HDV cleavage reaction occurs spontaneously in the reaction conditions required for in vitro transcription. The cleaved tRNA product was then purified from a denaturing polyacrylamide gel using the crush and soak method. Removal of the 2′, 3′-cyclo-phosphate group on the 3′ end of the purified tRNA product was performed by T4 polynucleotide kinase (NEB). The repaired tRNA product was quantified using a Qubit fluorometer and individual tRNA species were mixed to cover approximately 3.5 orders of magnitude. Appropriate mixing of the spike-in mix was assessed with a Qubit Fluorometer (Thermo Fisher) and Bioanalyzer (Agilent). Seventeen nanograms of control tRNAs were spiked into 1 µg of total RNA from HEK293 total RNA treated with demethylase.

### QuantM-tRNA-seq library preparation

Total RNA samples were quantified using a nanodrop spectrophotometer (Thermo Fisher) prior to library preparation and RNA integrity was checked on a 1.2% denaturing formaldehyde agarose gel. To remove 3′ conjugated amino acids, total RNA was deacylated at 37 °C for 45 minutes in deacylation buffer (final concentration 20 mM Tris-HCL pH = 9.0) at a final concentration of 1 µg/µL. Where indicated, deacylated total RNA was treated with demethylase (rtStarTM tRNA-optimized First-Strand cDNA Synthesis Kit, ArrayStar) and cleaned up per the manufacturer’s instructions. One microgram of deacylated total RNA from each sample was subject to library preparation. Ten picomole of the 3′ and 10 pmol of the 5′ single-stranded adapter mix (2.5 pmol of each adapter 5′-TGrGrA-3′, 5′-TGrGrT-3′, 5′-TGrGrG-3′, 5′-TGrGrC-3′; Supplementary Data 1) were added to a 200 µL thin-walled amplification tube and denatured at 95 °C for 2 min. Then annealing buffer was added to a final concentration of 5 mM Tris-HCl (pH 8.0), 0.5 mM ethylenediaminetetraacetic acid (EDTA), and 10 mM MgCl_2_ and incubated at 37 °C for 15 min to hybridize the annealed double-stranded adapter to tRNA. The ligation reaction was catalyzed by 5 U/µL of RNA ligase 2 (NEB) with the manufacturer’s suggested conditions at 37 °C for 60 min then 4 °C at 60 min. All reactions were ethanol precipitated with glycoblue (Thermo Fisher) followed by two 75% ethanol washes, then suspended in 10 µL of dH_2_O. Following ligation, synthesis of cDNA began with hybridization of the RT primer to the ligated total RNA with a final concentration of 0.5 pmol/µL (10 pmol total). The samples were incubated at 70 °C for 2 min and temperature was reduced to 37 °C by 0.1 °C/s. Synthesis of cDNA was achieved using Superscript IV at 55 °C for 60 min. To remove DNA-RNA dimers following cDNA synthesis, RNA was hydrolyzed with a final concentration of 0.1 N NaOH in dH_2_O at 98 °C for 20 min. All reactions were ethanol precipitated with glycoblue (Thermo Fisher) followed by two 75% ethanol washes, then suspended in 12 µL of dH_2_O. cDNA libraries were separated using 7 M urea 6% denaturing polyacrylamide gels. Gels were stained with 1× SYBR gold (Thermo Fisher) in 1× TBE for 15 min and regions representing tRNA derived cDNAs were excised on a UV light box. Gel slices were sheared through the bottom of a 0.5 mL tube nested in a 1.7 mL tube by centrifugation then suspended in 400 µL of DNA elution buffer (300 mM NaCl, 10 mM Tris-HCl (pH = 8.0), 1 mM EDTA), incubated on dry ice for 30 min, and allowed to incubate at room temperature overnight on a standing rotator. cDNA was isopropanol precipitated with glycoblue followed by two 75% ethanol washes then was resuspended in 12 µL of dH_2_O. Circularization of cDNA libraries was performed with CircLigase (Epicentre) at 0.5 U/µL using the manufacturer’s suggested conditions at 60 °C for 1 h. The reaction was terminated with incubation at 80 °C for 20 min. All reactions were ethanol precipitated with glycoblue followed by two 75% ethanol washes, then suspended in 12.5 µL of dH_2_O. cDNA libraries were amplified using the NEBnext Ultra II Q5 next-generation master mix (NEB) with the manufacturer’s suggested conditions. HEK293 libraries were amplified for seven cycles and mouse tissue libraries amplified for 7–9 cycles. Amplified libraries were gel purified from 2% agarose gels stained with 0.05 mg/mL ethidium bromide. Regions of interest (100–250 bp) were excised on a UV light box and purified using the Qiaquick gel extraction kit (Qiagen) taking care to dissolve gel slices at room temperature and using all optional steps. All libraries were ethanol precipitated with glycoblue (Thermo Fisher) with two 75% ethanol washes, then suspended in 10 µL of dH_2_O before submitting for sequencing. Library concentration was assessed using a Qubit (Thermo Fisher), quality was assessed on a DNA HS bioanalyzer chip (Agilent), and library multiplexing directed by qPCR. Sequencing was performed as single-end reads for 110 cycles on a NextSeq 550 (v2.5). All library QC, multiplexing, and sequencing was carried out by the Genomics Core Facility of the CWRU School of Medicine’s Genetics and Genome Sciences Department.

### Read quality control and alignment

Reads were first processed to remove 5′ adapter sequences using cutadapt --cut 2 then cutadapt -g TCCAACTGGATACTGGN -e 0.2 followed by cutadapt –a CCAGTATCCAGTTGGAATT -e 0.2 to remove 3′ CCA and adapter sequences. Custom human or mouse tRNA references were generated by collapsing identical tRNA sequences from gtRNAdb Release 18 hg38 or mm10 high-confidence mature tRNA fasta files. Mapping of human or mouse reads with the corresponding reference was done with bowtie2 using the parameters: --quiet --min-score G,1,8 --local -D 20 -R 3 -N 1 -L 10 -i S,1,0.5. Isodecoder-level read count tables for further analyses were produced by counting reads with MAPQ > 10 over reference tRNAs using the Rsubread package’s featureCounts function in R. Anticodon-level read count tables were then created by summing reads from all isodecoders with the same anticodon. In addition to raw read count tables, tables of both isodecoder and anticodon-level RPM mapped read values were generated by dividing raw read counts * 1,000,000 by the number of reads mapped.

### Differential expression analysis

The raw read count tables at both the anticodon-level and isodecoder-level across all seven mouse tissues described in the previous section were next used to perform differential tRNA expression analysis. The likelihood ratio test was applied to these tables using DESeq2 in R as detailed in https://hbctraining.github.io/DGE_workshop/lessons/08_DGE_LRT.html (Command: DESeq(raw_count_table, test = “LRT”, reduced = ~ 1)) using default settings and *p* value adjustment (Benjamin–Hochberg correction). Downstream data visualization and plotting were performed using ggplot2, gplots (heatmap.2), ggrepel, and ggforce in custom R scripts.

### Variant analysis

In order to analyze variants in tRNA sequencing reads, a custom Python script was used to generate variant counts at each position in every tRNA across all seven mouse tissues. In brief, bam files were read into the script and the CIGAR string and MD tags for each read were used to tabulate each mutation, insertion, or deletion across every ribonucleotide base of all tRNA in the mouse reference. In addition, 5′ ends of reads internal to tRNA were used to infer sites of RT stalling or fall-off. These four types of variants were summed at each position of each tRNA for a total variant count, and then a read coverage at each position was also calculated.

To identify significantly changed sequencing variants across tissues, we performed DEXseq analysis on the raw variant counts table in R. DEXseq was originally devised to identify alternative processing events in mRNA, but we reasoned that co-transcriptional splicing is similar in principle to RT-mediated misincorporation/stalling at RNA modifications. To ensure robust detection of variants that change across tissues, we added two additional filtering steps. First, for a given tRNA base, we required that variant percentage be >1% on average in every tissue. Next, we only accepted base-level variants that changed variant percentage at least 1.5-fold across tissues. Downstream data visualization and plotting were performed using ggplot2, gplots, ggrepel, and ggforce in custom R scripts as well as matplotlib in custom python scripts.

### Calculation of nucleotide frequencies across CircLigase junction

For each band from the cDNA gel depicted in Fig. 2d, we can reasonably assume that the majority of the stalling resulting in the shortest cDNA ends in A (m^1^A)^23^. We also know that longest full-length cDNA will end in T, as the 5′ adapter ends in T. The shortest full-length cDNA (without 5′ adapter) is also likely to end in T, as this is complementary to the discriminator base, which is highly skewed toward A^40–42^. For all other minor bands, we do not know what base RT is stalling over, so our prior estimate was 25% for each base. Given these prior parameters and the amounts of each cDNA species from Fig. 2d and library dsDNA in our Bioanalyzer trace (Source Data), we can calculate a predicted nucleotide frequency for the 3′ position depicted in Fig. 2e. For reads, we simply calculated nucleotide frequencies of the first three bases across all reads.

### Reporting summary

Further information on research design is available in the Nature Research Reporting Summary linked to this article.

## Supplementary information

Supplementary Information

Reporting Summary

Description of Additional Supplementary Files

Supplementary Software

Supplementary Data 1

Supplementary Data 2

## Data Availability

The data that support this study are available from the corresponding author upon reasonable request. The datasets generated during and/or analyzed during the current study are all available in the NCBI Gene Expression Omnibus repository with accession number GSE141436. Source data are provided with this paper.

## Source data

Source Data

## Code availability

Code used to perform the analyses presented in Figs. 1–5 and all Supplementary figures utilized only published packages for R that are detailed in “Methods”. Custom code for the analyses presented in Fig. 6 was written in Python. All code is provided as a Supplementary Software file.
