# Supplementary material for: Quantitative tRNA-sequencing uncovers metazoan tissue-specific tRNA regulation
Source: Nat Commun. 2020 Aug 14;11:4104. doi: 10.1038/s41467-020-17879-x (PMC7428014; doi:10.1038/s41467-020-17879-x)
Supplement: Supplementary file 1 — Supplementary Information [file 41467_2020_17879_MOESM1_ESM.pdf]

Pinkard et al Supplementary Figure 1

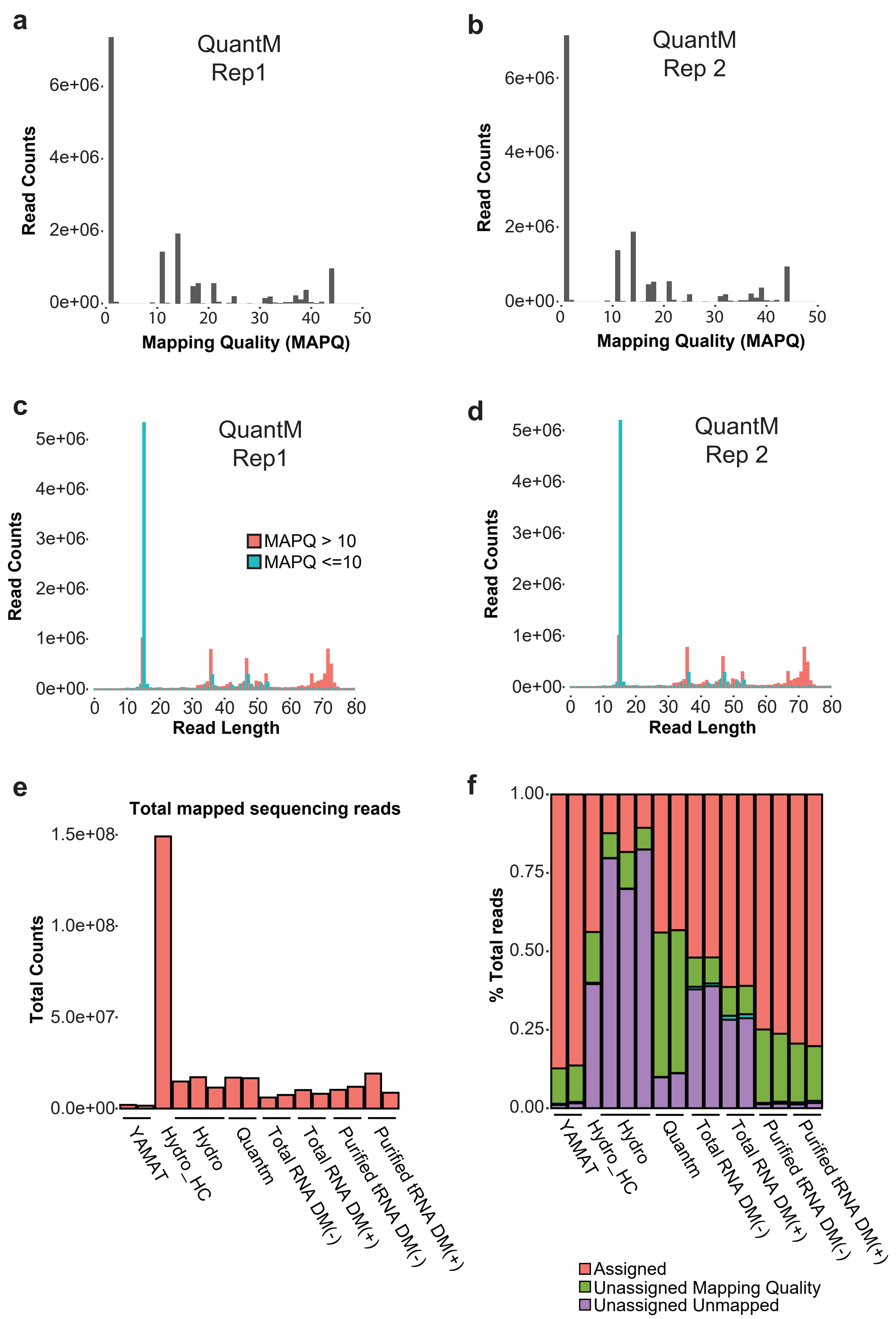

Pinkard et al Supplementary Data Figure 2

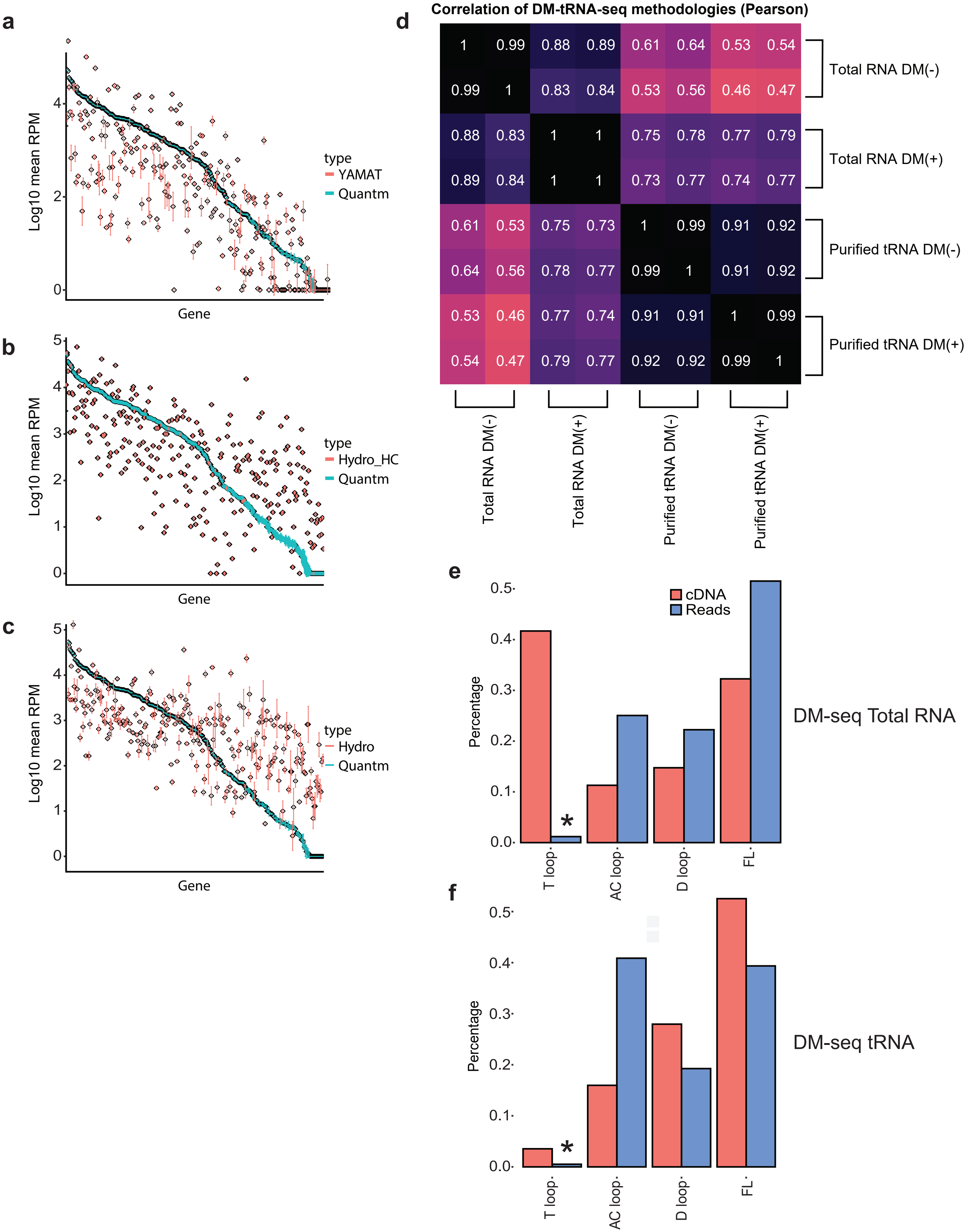

Pinkard et al Supplementary Figure 3

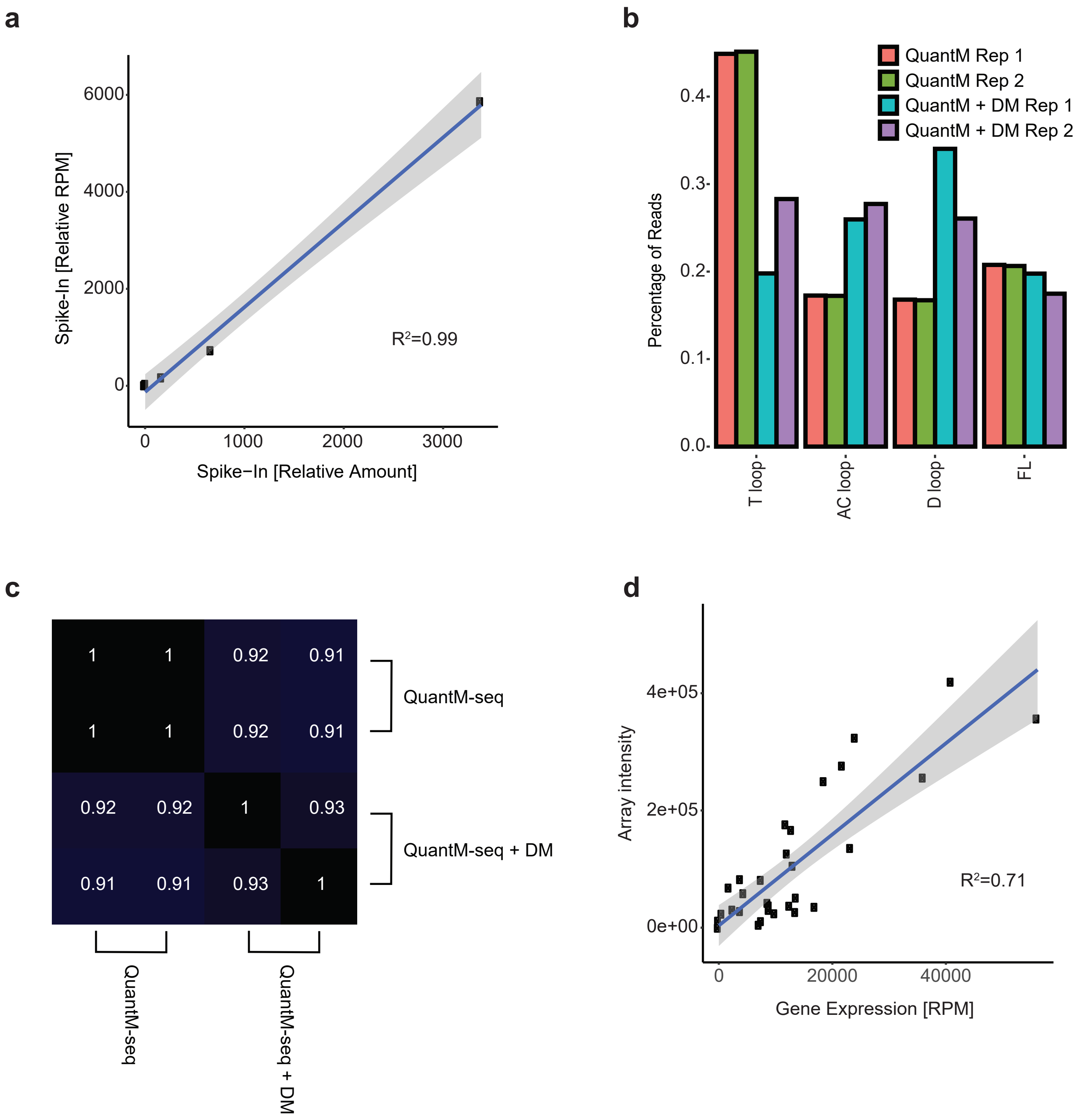

Pinkard et al Supplementary Figure 4

a

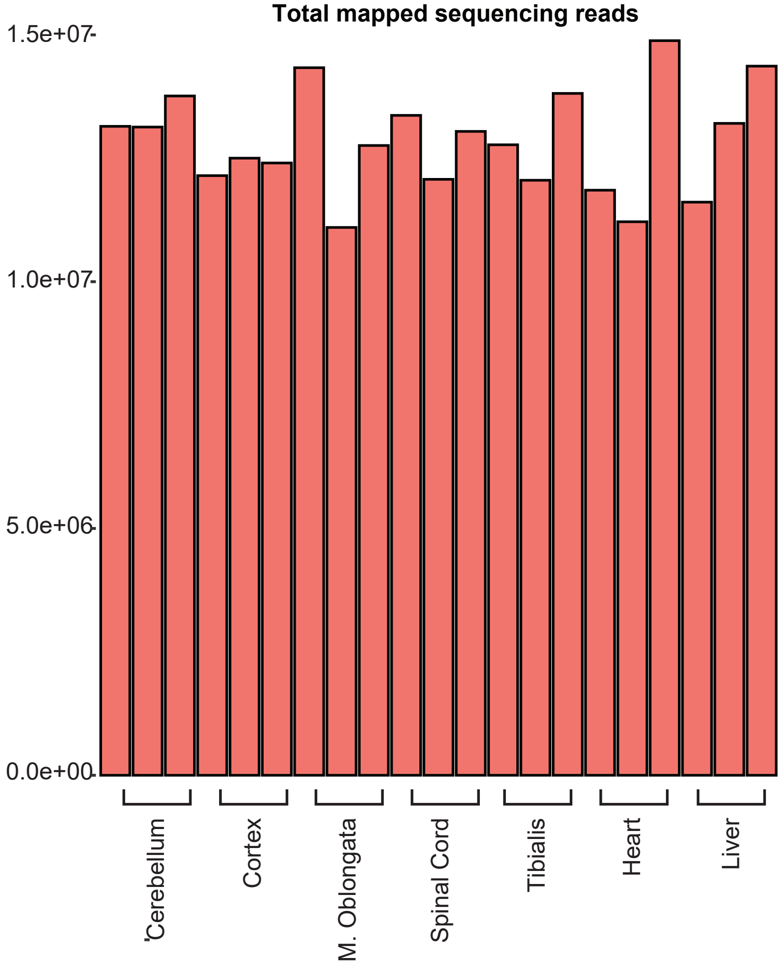

b

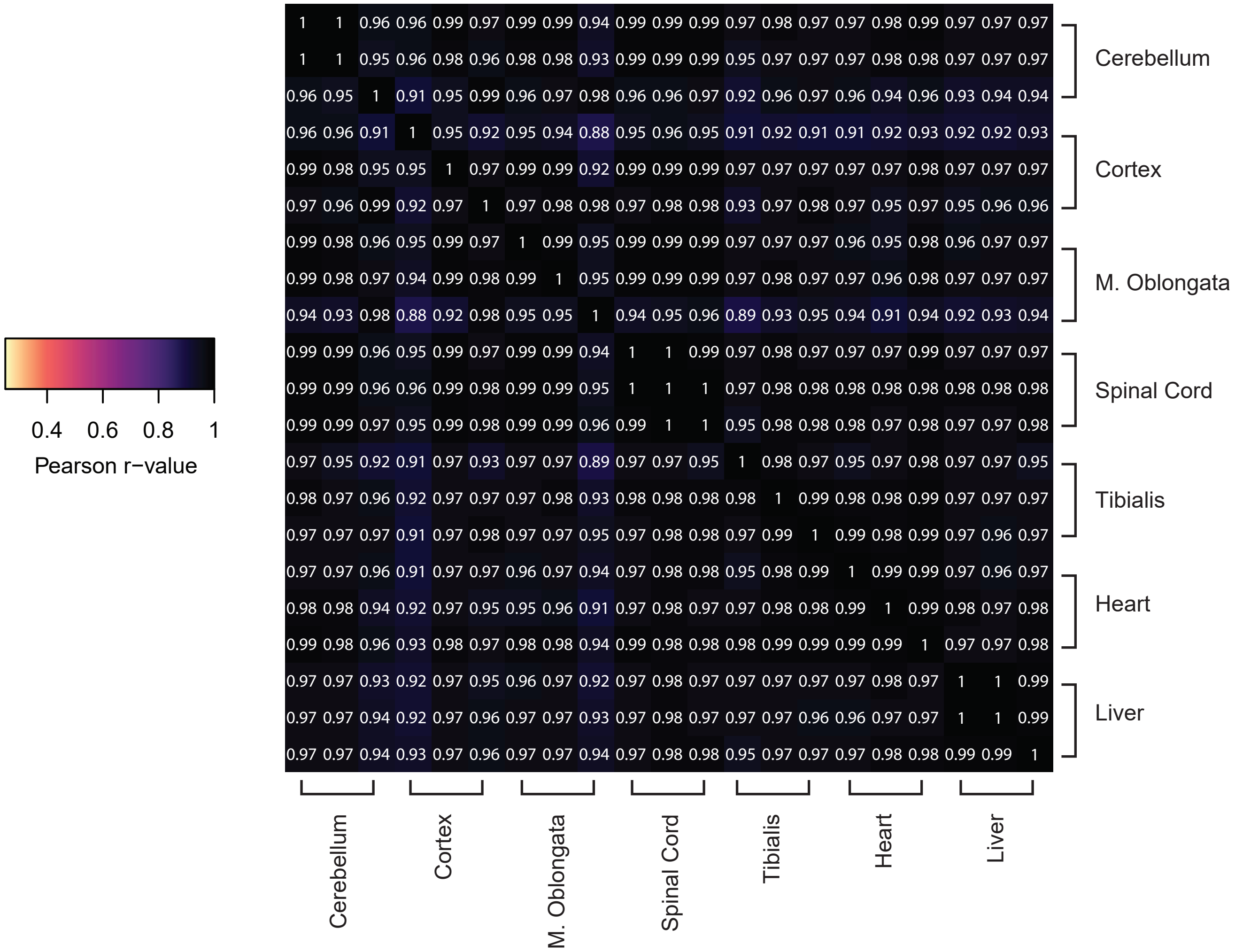

Pinkard et al Supplementary Figure 5

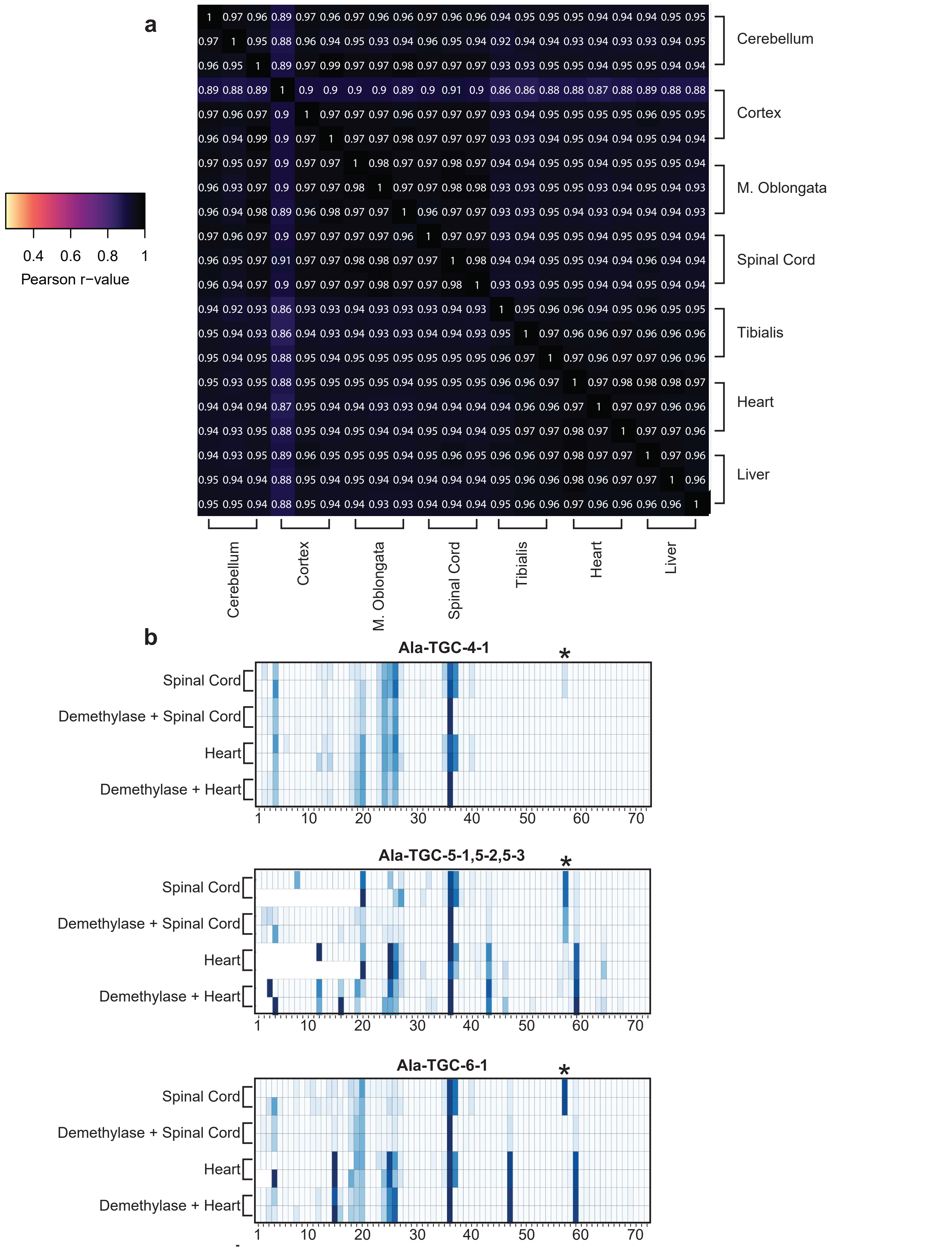

Supplementary Figure Legends

Supplementary Figure 1: Sequencing metrics of QuantM-seq from HEK293 cells. Read count histograms of mapping quality scores (MAPQ) derived from QuantM-seq of HEK 293 cells for biological replicate 1 (**a**) and biological replicate 2 (**b**). Bar charts of read lengths vs. read counts classified as MAPQ > 10 or MAPQ <= 10 for QuantM-seq of HEK 293 cells for biological replicate 1 (**c**) and biological replicate 2 (**d**). **e,** A histogram depicting the total mapped reads (MAPQ > 10) for YAMAT-seq (YAMAT), Hydro-tRNA-seq with high coverage (Hydro_HC), Hydro-tRNA-seq with lower coverage (Hydro), QuantM-tRNA-seq (QuantM), DM-tRNA-seq performed on PAGE purified tRNA +/- pretreatment with demethylase (Total RNA DM +/-), DM-tRNA-seq performed on total RNA +/- pretreatment with demethylase (Purified tRNA DM +/-). Each bar represents a separate biological replicate for each methodology performed on HEK293 cells. **f,** Fraction of reads Assigned (MAPQ > 10), Unassigned Mapping Quality (MAPQ <= 10), or unmapped. Each bar represents a separate biological replicate for each methodology performed on HEK293 cells.

Supplementary Figure 2: Comparison of QuantM-seq with other tRNA-seq protocols, including a high output Hydro-seq library. Plots of $\log_{10}$-transformed reads per million for unique tRNA sequences. QuantM-seq is always represented by cyan dots compared to YAMAT-seq (N=2 biological replicates) (**a**), Hydro_HC high coverage hydro-seq library (N=1 sample) (**b**), or lower coverage Hydro-seq libraries (N=3 biological replicates) (**c**) in red. Error bars represent standard deviation of

replicates. **d,** Pearson correlation coefficients between gene-level expression for each dataset. Total RNA DM(-): DM-tRNA-seq performed on total RNA without demethylase treatment, Total RNA DM(+): DM-tRNA-seq performed on total RNA with demethylase treatment, Purified tRNA DM(-): DM-tRNA-seq performed on gel purified tRNA without demethylase treatment, Purified tRNA DM(+): DM-tRNA-seq performed on gel purified tRNA with demethylase treatment. Bar charts of percentage of reads from DM-tRNA-seq on total RNA (**e**) or purified tRNA (**f**) where reverse transcriptase stalls or falls of in the T-loop, anticodon (AC) loop, D-loop, or at the end of tRNA (full length; FL). Red and Blue bars represent two separate biological replicates. cDNA represents values derived from densitometry of cDNA gels, reads are values from sequencing reads. * - denotes short reads (15 nt) that represent stalling at m1A in the T-loop clearly evident in cDNA were discarded from the GEO record.

Supplementary Figure 3: Demethylase treatment prior to QuantM-seq does not detectably improve quantitation. a, Scatter plot of average relative amount of *in vitro* transcribed *E. coli* tRNAs spiked into two HEK293 QuantM-seq libraries versus amounts detected by sequencing in reads per million. Shaded area represents the 95% confidence interval of the linear trendline. **b,** Bar chart of percentage reads from QuantM-seq of RNA from HEK293 cells treated with or without demethylase (DM) where reverse transcriptase stalls or falls off in the T-loop, anticodon (AC) loop, D-loop, or at the end of tRNA (full length; FL). Each bar represents individual biological replicates. **c,** Pearson correlation coefficients between QuantM-seq libraries with and without demethylase treatment (DM - demethylase). **d,** Scatter plot of expression values

from QuantM-seq performed on HEK293 RNA treated with demethylase versus tRNA array intensities. Shaded area represents the 95% confidence interval of the linear trendline.

Supplementary Figure 4: QuantM-seq of RNA from mouse tissues. a, Bar graph depicting sequencing depth for each mouse tissue tRNA dataset analyzed. **b,** Pearson correlation coefficients for isodecoder-level expression between each mouse tissue.

Supplementary Figure 5: Variants in sequencing change across tissues in tRNA bases known to be modified. a, Pearson correlation coefficients between isodecoder-level variant fractions for each mouse tissue. Only tRNA bases with variant fractions that were >1% in all tissue samples were considered as potential sequence variants. **b,** Heatmaps for three isodecoders representing variant fractions at each tRNA position (x-axis) across tissue samples (y-axis). RNA from a CNS tissue (spinal cord) and a non-CNS tissue (heart) were either treated or untreated with demethylase before QuantM-seq. Note that position 56 (*), which varies only in CNS tissues (Fig. 6), loses variation upon treatment with demethylase, indicating that the m^1A was methyl group was removed. The numbers below the plot indicate nucleotide position.
