# Supplementary material for: Quantitative tRNA-sequencing uncovers metazoan tissue-specific tRNA regulation
Source: Nat Commun. 2020 Aug 14;11:4104. doi: 10.1038/s41467-020-17879-x (PMC7428014; doi:10.1038/s41467-020-17879-x)
Supplement: Supplementary file 3 — Description of Additional Supplementary Files [file 41467_2020_17879_MOESM3_ESM.pdf]

Description of Additional Supplementary Files

File name: Supplementary Data 1
Description: details the exact oligonucleotide sequences utilized in the manuscript. This includes the 30 sequences used in the hybridization based arrays and northern blots in figures 1d-f. the last columns describe the usage of the sequence in either the 30 probe array only or the both the array and northern blotting experiments. In addition, Supplementary Table 1 contains sequences used throughout the QuantM-tRNA-seq protocol. The [3' AD] and [5' AD] adapter sequences used in the ligation step, the [RT primer] sequence used for cDNA synthesis, and the common [PCR primer] used in all amplification reactions with the [PCR barcoded primer] containing an Illumina barcode for multiplexing libraries. For the spike in control, we included the five e. coli tRNA genes in vitro transcribed containing a T7 RNA polymerase promoter sequence and HDV ribozyme sequence.

File name: Supplementary Data 2
Description: contains in depth information and analysis pertaining to the 30 oligonucleotide sequences utilized in both the northern blot and array experiments. 'Complement' gives the complementary sequence to the probe. The table contains various metrics which may be useful for other research groups. [Probe] describes the set of tRNA genes predicted to hybridize to the oligonucleotide sequence. [Average array intensity] is the background subtracted array intensity for each probe averaged from triplicate experiments. The four [Array x 'metric'] columns describe the correlation of the various metrics (GC content, melting temperature, predicted folding structure, and temperature of melting for the double stranded DNA sequence) to the average array intensity.

File name: Supplementary Code 1
Description: Supplementary code contains all files necessary for reproduction the analyses performed and displayed within the figures of the manuscript. [tRNA_processing_script.txt] contains the bash script necessary for tRNA genome generation (either human or mouse), read trimming and alignment to the appropriate tRNA genome. The [HEK293_human.rmd] and [tissue_mouse.rmd] files contain the R code necessary for differential expression analysis of individual tRNA genes, tRNA genes summed into anticodon families, and variant levels. In addition, these files contain all code necessary to generate preliminary figures presented in the manuscript. Each code chunk corresponds to a single main text or supplementary figure. The [.ipynb] contain python code necessary for producing the appropriate preliminary tables for variant analysis. Starting with .fastq files, these preliminary tables can be utilized in the [.rmd] files for producing both the main text and supplementary figures relating to variant analysis.
