# Supplementary material for: Quantitative tRNA-sequencing uncovers metazoan tissue-specific tRNA regulation
Source: Nat Commun. 2020 Aug 14;11:4104. doi: 10.1038/s41467-020-17879-x (PMC7428014; doi:10.1038/s41467-020-17879-x)

Ladder ispuc18 digested with
hindIII then end labled with
T4 PNK gamma ATP

gene	northern intensity	northern bkg subtracted
Arg-ACG	502728.56	459236.99
Lys-CTT	376777.36	333285.79
Lys-TTT	232481.41	188989.84
Glu-TTC	138106.32	94614.75
Cys-GCA	201572.9	158081.33
Arg-CCG	121670.31	78178.74
Ser-CGA	114678.45	71186.88
Tyr-GTA	57687.99	14196.42
Met-CAT	103463.11	59971.54
Tyr-ATA	44397.59	906.02

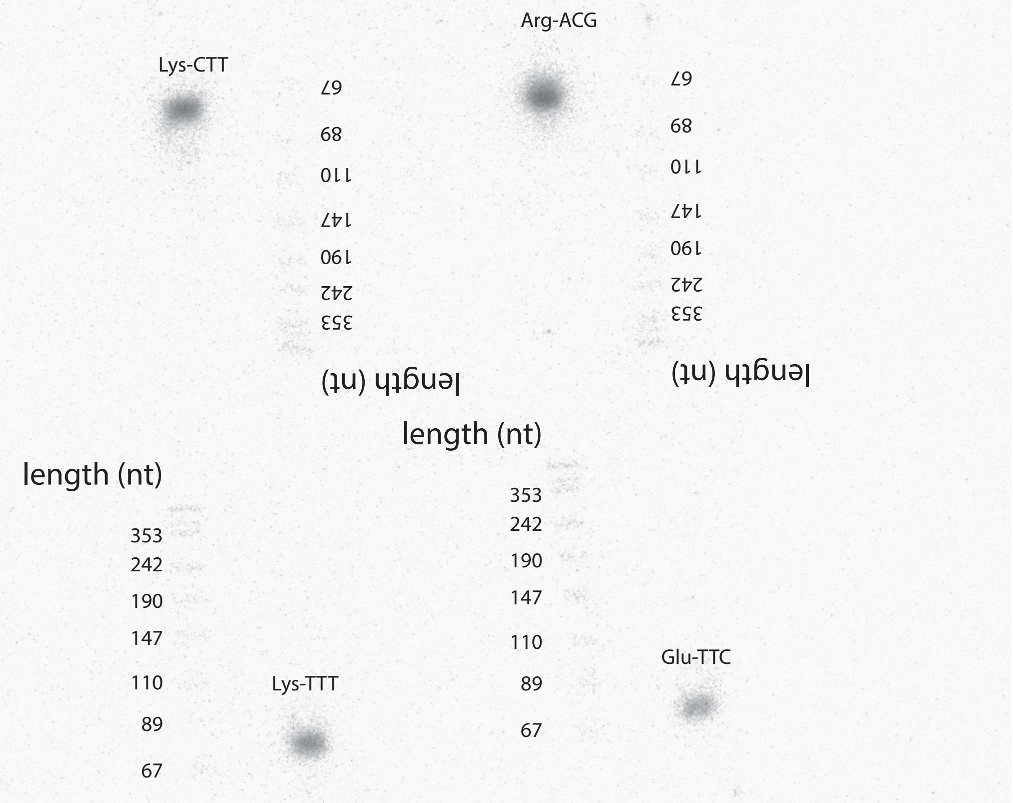

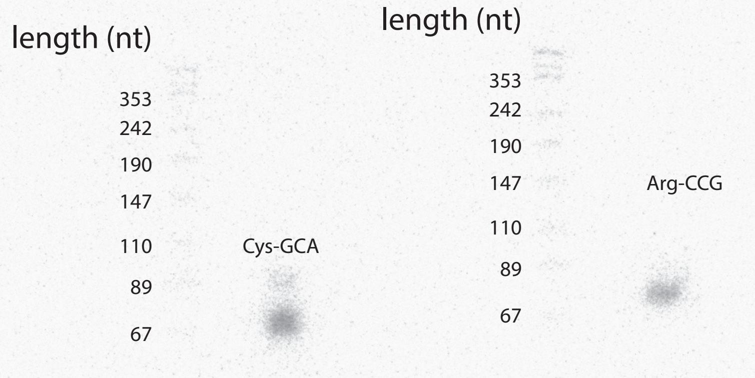

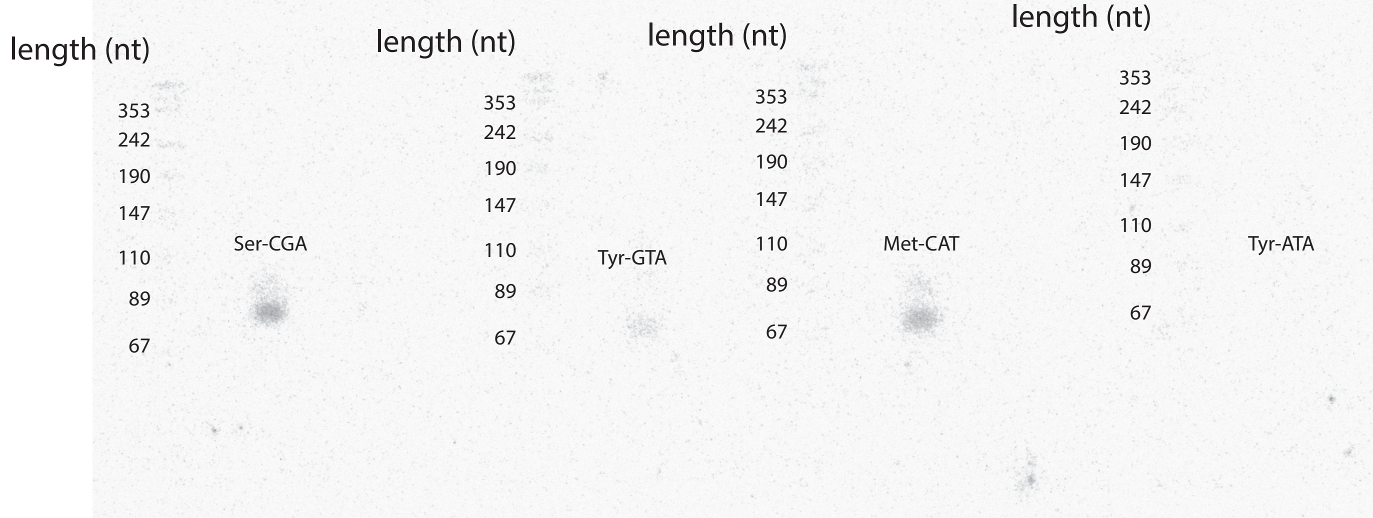

Supplement: Supplementary file 7 — Source Data [file 41467_2020_17879_MOESM7_ESM.zip › Source Data/Annotated pdf/figure 1f annotated.pdf]
