# Supplementary material for: Quantitative tRNA-sequencing uncovers metazoan tissue-specific tRNA regulation
Source: Nat Commun. 2020 Aug 14;11:4104. doi: 10.1038/s41467-020-17879-x (PMC7428014; doi:10.1038/s41467-020-17879-x)
Supplement: Supplementary file 7 — Source Data [file 41467_2020_17879_MOESM7_ESM.zip › Source Data/Raw tif/Pinkard_et_al_2020_bioanalyzer_source_data.pdf]

Assay Class: High Sensitivity DNA Assay   Created: 2/12/2019 1:07:17 PM
Data Path: C:\...gh Sensitivity DNA Assay_DE04103509_2019-02-12_13-07-17.xad   Modified: 2/12/2019 2:16:22 PM

Electrophoresis Assay Details

General Analysis Settings

Number of Available Sample and Ladder Wells (Max.) : 12
Minimum Visible Range [s] : 32
Maximum Visible Range [s] : 138
Start Analysis Time Range [s] : 33
End Analysis Time Range [s] : 137.5
Ladder Concentration [pg/µl] : 1950
Uses Standard Area for Ladder Fragments
Lower Marker Concentration [pg/µl] : 125
Upper Marker Concentration [pg/µl] : 75
Used Upper Marker for Quantitation
Standard Curve Fit is Point to Point
Show Data Aligned to Lower and Upper Marker

Integrator Settings

Integration Start Time [s] : 33.05
Integration End Time [s] : 137
Slope Threshold : 0.8
Height Threshold [FU] : 5
Area Threshold : 0.1
Width Threshold [s] : 0.6
Baseline Plateau [s] : 0.5

Filter Settings

Filter Width [s] : 0.5
Polynomial Order : 4

Ladder

Ladder Peak	Size	Area
1	35	160
2	50	210
3	100	208
4	150	221
5	200	242
6	300	270
7	400	305
8	500	306
9	600	336
10	700	321
11	1000	366
12	2000	413
13	3000	411
14	7000	400
15	10380	214

© Copyright 2003 - 2017 Agilent Technologies, Inc.

Assay Class: High Sensitivity DNA Assay
Data Path: C:\...gh Sensitivity DNA Assay_DE04103509_2019-02-12_13-07-17.xad

Created: 2/12/2019 1:07:17 PM
Modified: 2/12/2019 2:16:22 PM

Electropherogram Summary Continued ...

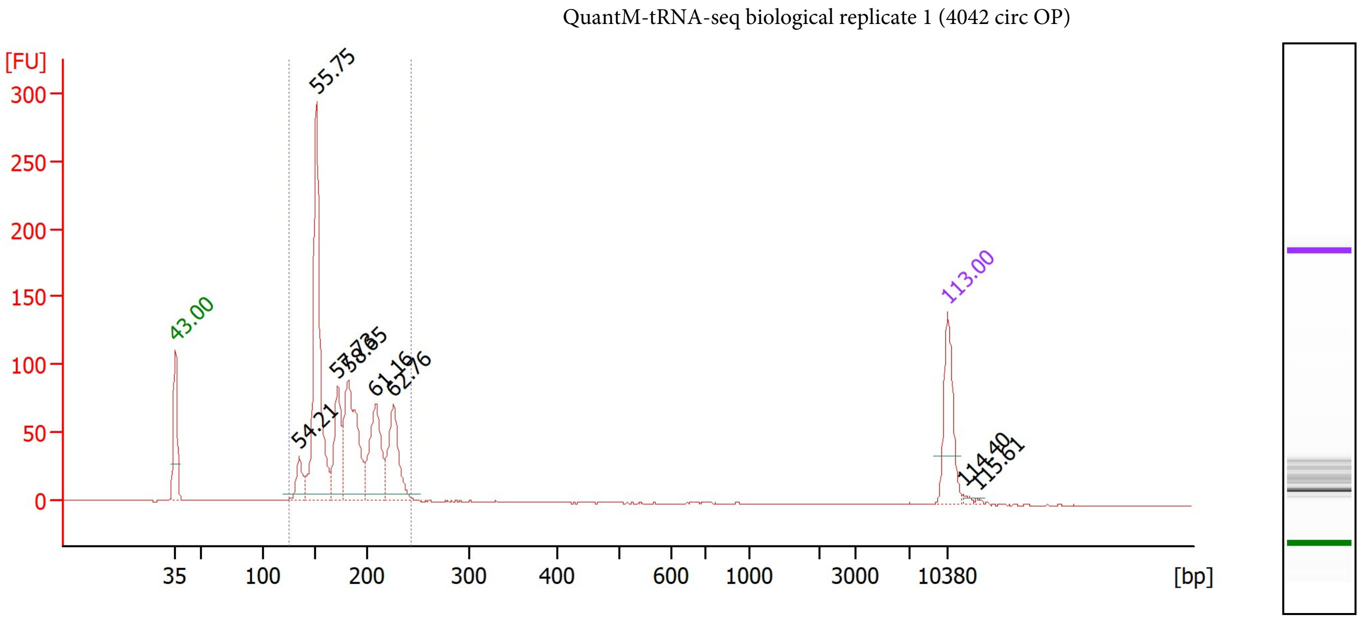

Overall Results for sample 3 : QuantM-tRNA-seq biological replicate 1 (4042 circ OP)

Number of peaks found: 8
Corr. Area 1: 1,034.4
Noise: 0.3

Peak table for sample 3 :

Peak		Size [bp]	Conc. [pg/µl]	Molarity [pmol/l]	Observations
1	◀	35	125.00	5,411.3	Lower Marker
2		134	48.76	549.6	
3		151	449.56	4,520.3	
4		172	128.56	1,135.6	
5		181	234.67	1,962.4	
6		208	156.33	1,139.6	
7		225	134.95	908.2	
8	▶	10,380	75.00	10.9	Upper Marker
9		11,764	0.00	0.0	
10		12,957	0.00	0.0	

Region table for sample 3 : QuantM-tRNA-seq biological replicate 1 (4042 circ OP)

From [bp]	To [bp]	Corr. Area	% of Total	Average Size [bp]	Size distribution in CV [%]	Conc. [pg/µl]	Molarity [pmol/l]	Co lor
125	243	1,034.4	97	179	15.8	1,158.12	10,114.1	

© Copyright 2003 - 2017 Agilent Technologies, Inc.

Assay Class: High Sensitivity DNA Assay
Data Path: C:\...gh Sensitivity DNA Assay_DE04103509_2019-02-12_13-07-17.xad

Created: 2/12/2019 1:07:17 PM
Modified: 2/12/2019 2:16:22 PM

Electropherogram Summary Continued ...

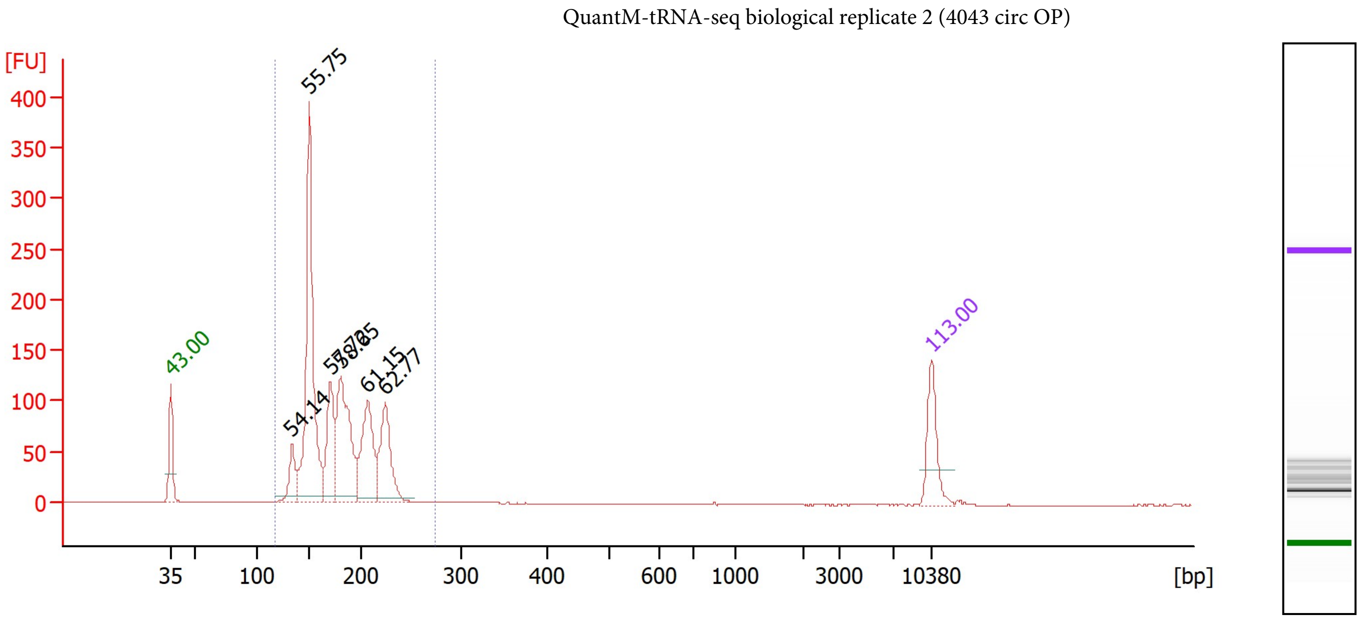

Overall Results for sample 4 : QuantM-tRNA-seq biological replicate 1 (4043 circ OP)

Number of peaks found:	6	Corr. Area 1:	1,492.7
Noise:	0.4		

Peak table for sample 4 :

Peak		Size [bp]	Conc. [pg/µl]	Molarity [pmol/l]	Observations
1	◀	35	125.00	5,411.3	Lower Marker
2		134	80.52	912.8	
3		151	610.19	6,135.3	
4		171	175.72	1,553.9	
5		181	316.91	2,650.8	
6		208	211.57	1,543.4	
7		225	180.84	1,216.4	
8	▶	10,380	75.00	10.9	Upper Marker

Region table for sample 4 : QuantM-tRNA-seq biological replicate 1 (4043 circ OP)

From [bp]	To [bp]	Corr. Area	% of Total	Average Size [bp]	Size distribution in CV [%]	Conc. [pg/µl]	Molarity [pmol/l]	Color
118	275	1,492.7	99	178	16.2	1,585.95	13,915.7	

 © Copyright 2003 - 2017 Agilent Technologies, Inc.

Assay Class:	High Sensitivity DNA Assay	Created:	2/12/2019 1:07:17 PM
Data Path:	C:\...gh Sensitivity DNA Assay_DE04103509_2019-02-12_13-07-17.xad	Modified:	2/12/2019 2:16:22 PM

Electropherogram Summary Continued ...

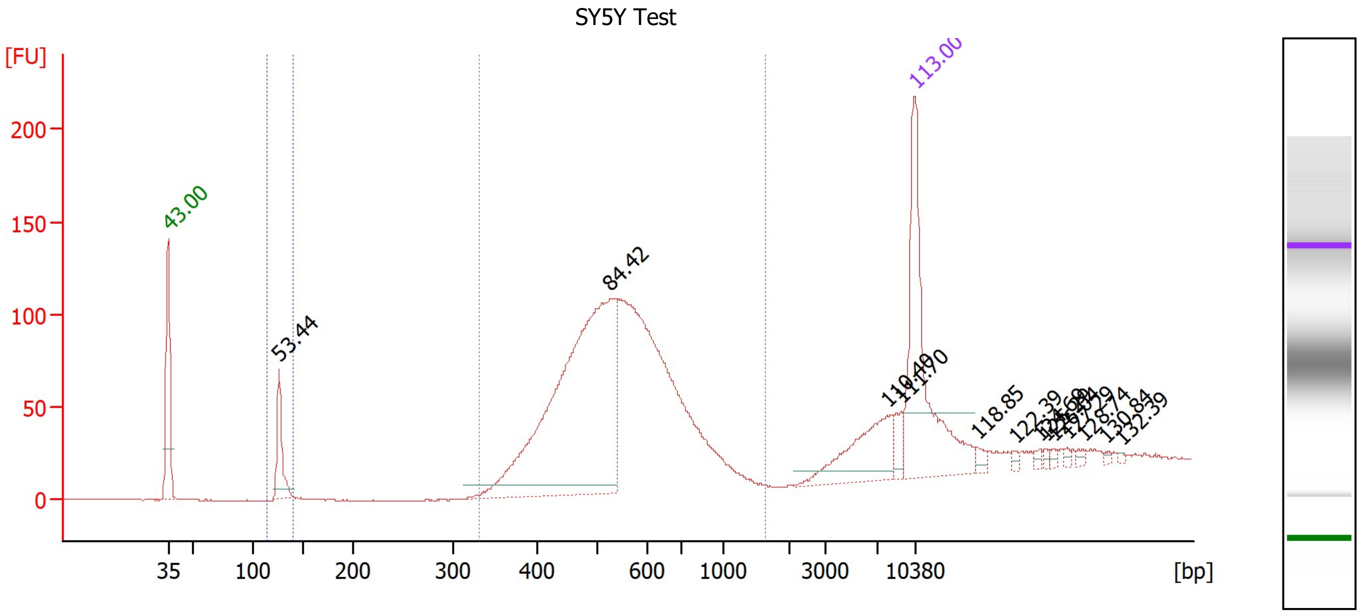

Overall Results for sample 5 : SY5Y Test

Number of peaks found:	13	Corr. Area 1:	1,361.4
Noise:	0.4	Corr. Area 2:	59.6

Peak table for sample 5 : SY5Y Test

Peak		Size [bp]	Conc. [pg/µl]	Molarity [pmol/l]	Observations
1	◀	35	125.00	5,411.3	Lower Marker
2		126	33.95	407.0	
3		524	260.37	753.1	
4		7,813	36.43	7.1	
5		9,097	6.61	1.1	
6	▶	10,380	75.00	10.9	Upper Marker
7		16,155	0.00	0.0	
8		19,659	0.00	0.0	
9		21,930	0.00	0.0	
10		22,522	0.00	0.0	
11		23,262	0.00	0.0	
12		24,496	0.00	0.0	
13		25,927	0.00	0.0	
14		28,000	0.00	0.0	
15		29,531	0.00	0.0	

Region table for sample 5 : SY5Y Test

From [bp]	To [bp]	Corr. Area	% of Total	Average Size [bp]	Size distribution in CV [%]	Conc. [pg/µl]	Molarity [pmol/l]	Color
115	140	59.6	4	127	2.0	28.00	334.5	
333	1,619	1,361.4	83	553	21.4	434.40	1,252.7	

 © Copyright 2003 - 2017 Agilent Technologies, Inc.

Assay Class: High Sensitivity DNA Assay
Data Path: C:\...gh Sensitivity DNA Assay_DE04103509_2019-02-12_13-07-17.xad
Created: 2/12/2019 1:07:17 PM
Modified: 2/12/2019 2:16:22 PM

Gel Image

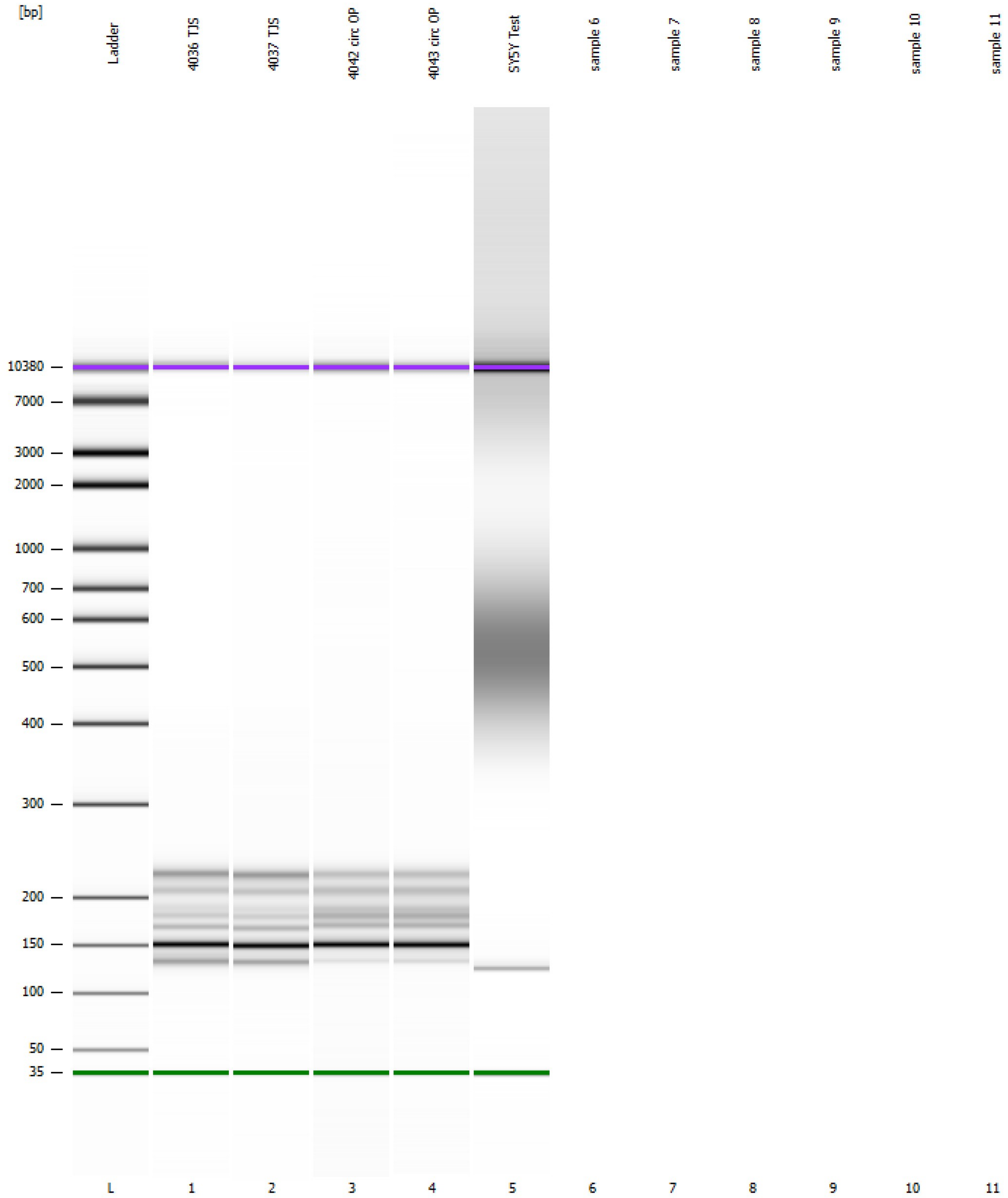

 © Copyright 2003 - 2017 Agilent Technologies, Inc.

Assay Class: High Sensitivity DNA Assay
Data Path: C:\...gh Sensitivity DNA Assay_DE04103509_2019-02-12_13-07-17.xad
Created: 2/12/2019 1:07:17 PM
Modified: 2/12/2019 2:16:22 PM

Curves

Standard Curve

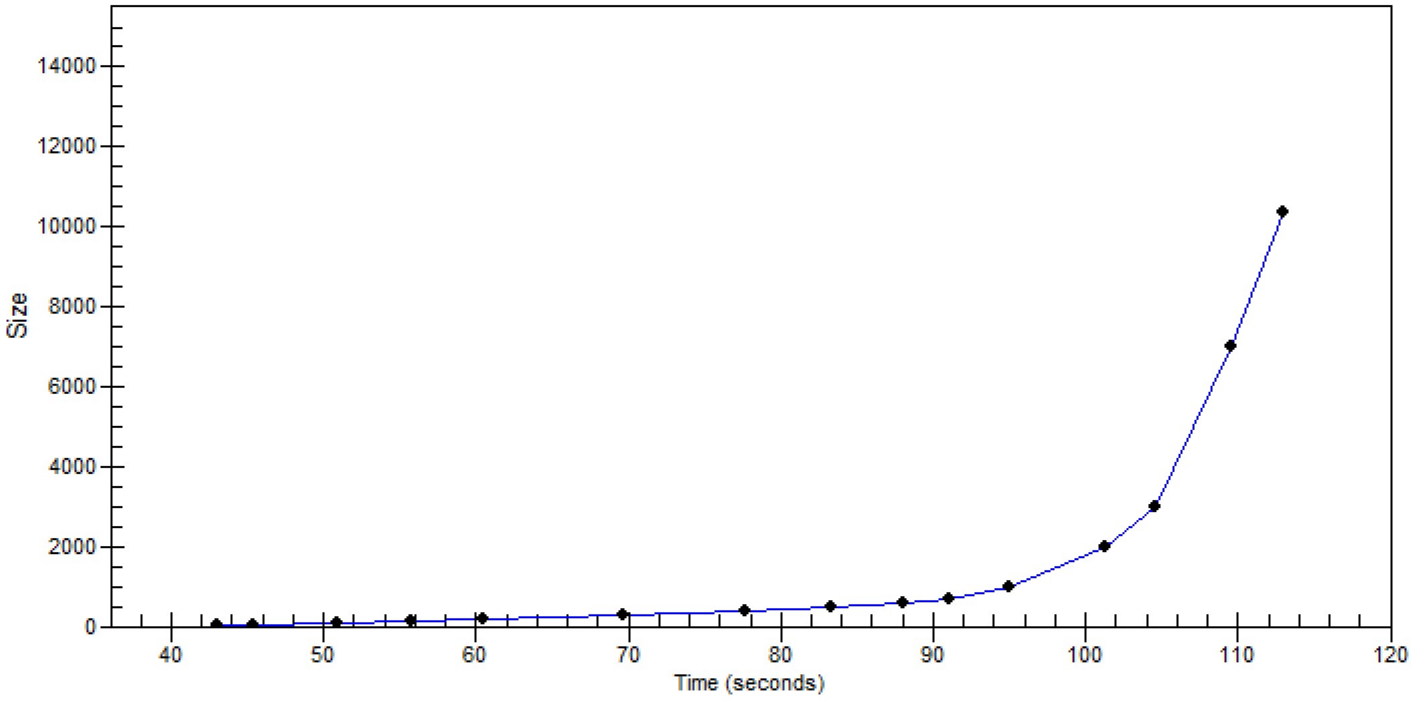

© Copyright 2003 - 2017 Agilent Technologies, Inc.
