# Supplementary figures and images for: Quantitative tRNA-sequencing uncovers metazoan tissue-specific tRNA regulation
Source: Nat Commun. 2020 Aug 14;11:4104. doi: 10.1038/s41467-020-17879-x (PMC7428014; doi:10.1038/s41467-020-17879-x)

Pinkard et al. 2020 Figure 1c

Ultra low range  
DNA Ladder (thermo)

length (nt)

300  
200  
150  
100  
75  
50  
35  
20  
10

This is what was used in figure

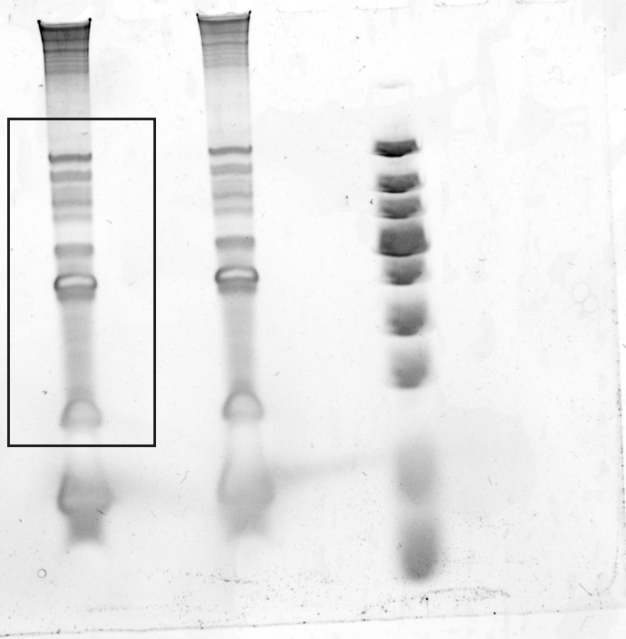

Supplement: Supplementary file 7 — Source Data [file 41467_2020_17879_MOESM7_ESM.zip › Source Data/Annotated pdf/figure 1c annotated.pdf]

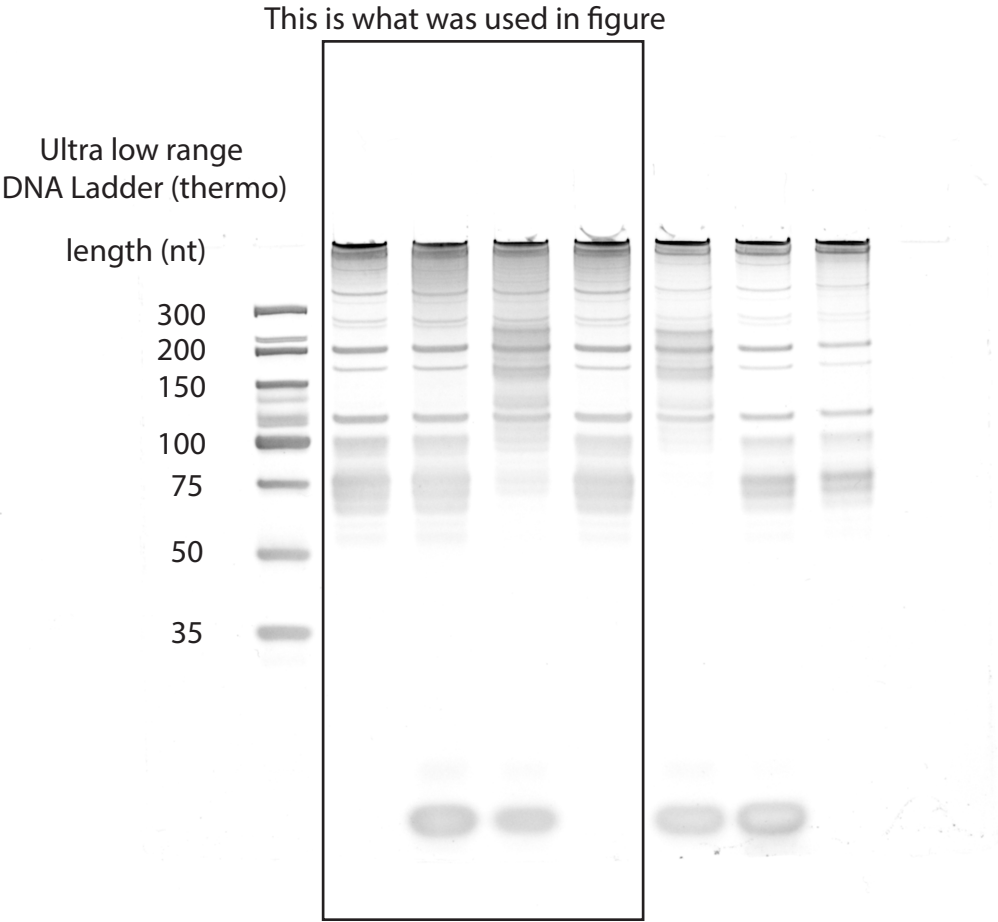

Supplement: Supplementary file 7 — Source Data [file 41467_2020_17879_MOESM7_ESM.zip › Source Data/Annotated pdf/figure 1b annotated.pdf]

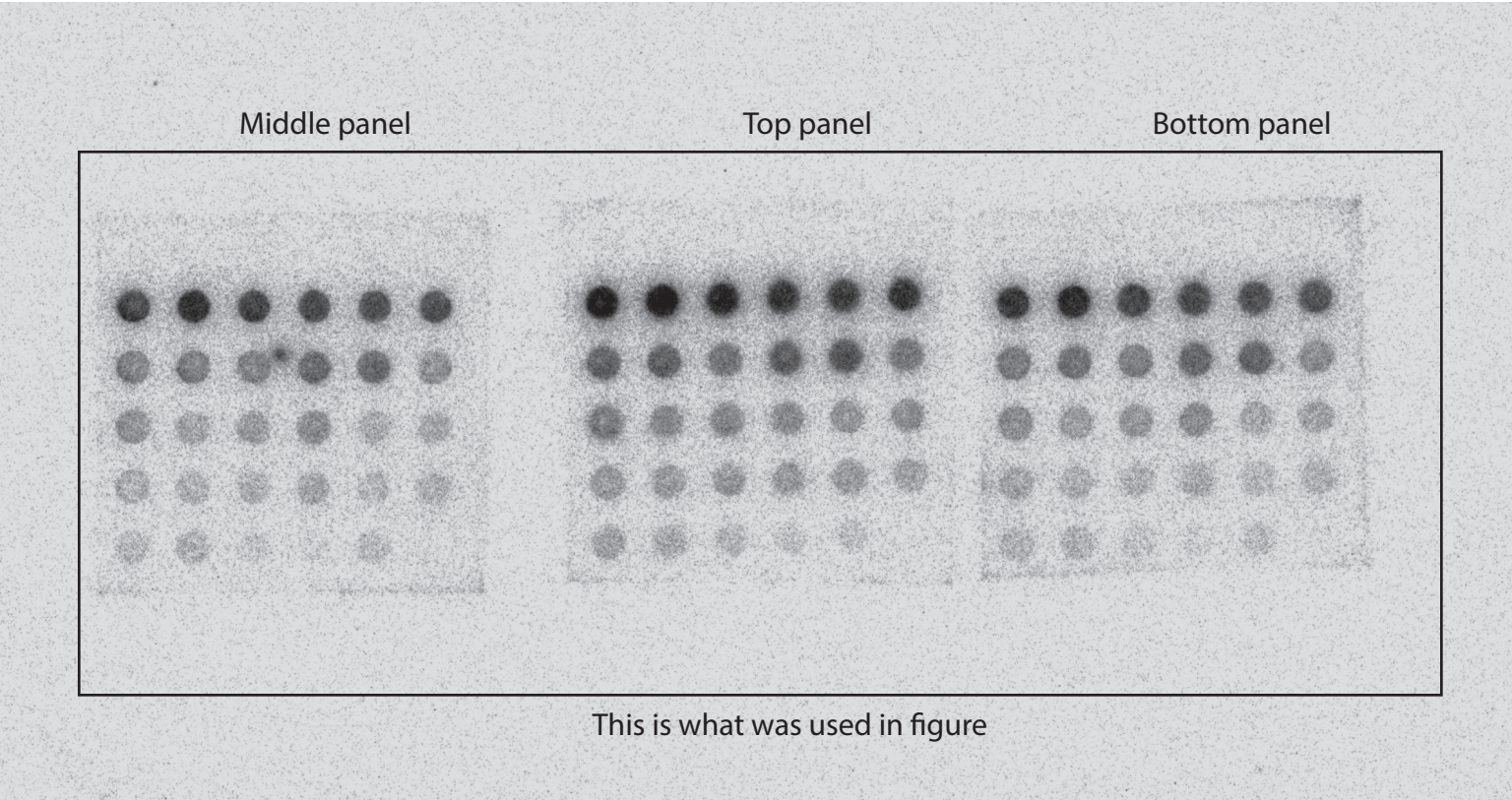

Supplement: Supplementary file 7 — Source Data [file 41467_2020_17879_MOESM7_ESM.zip › Source Data/Annotated pdf/figure 1d&e annotated.pdf]

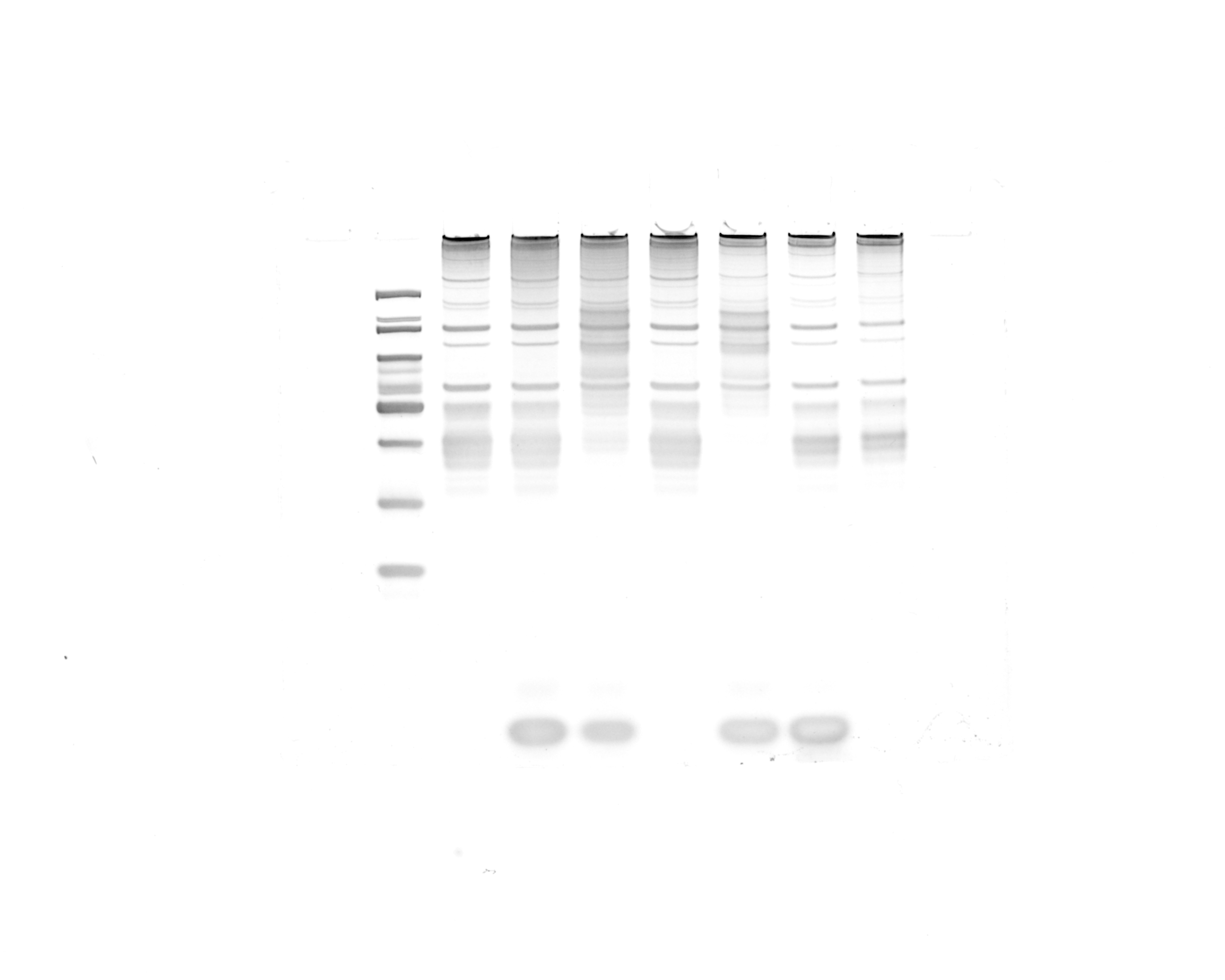

Supplement: Supplementary file 7 — Source Data [file 41467_2020_17879_MOESM7_ESM.zip › Source Data/Raw tif/Figure1b_RNL2_Ligation&controls.tif]

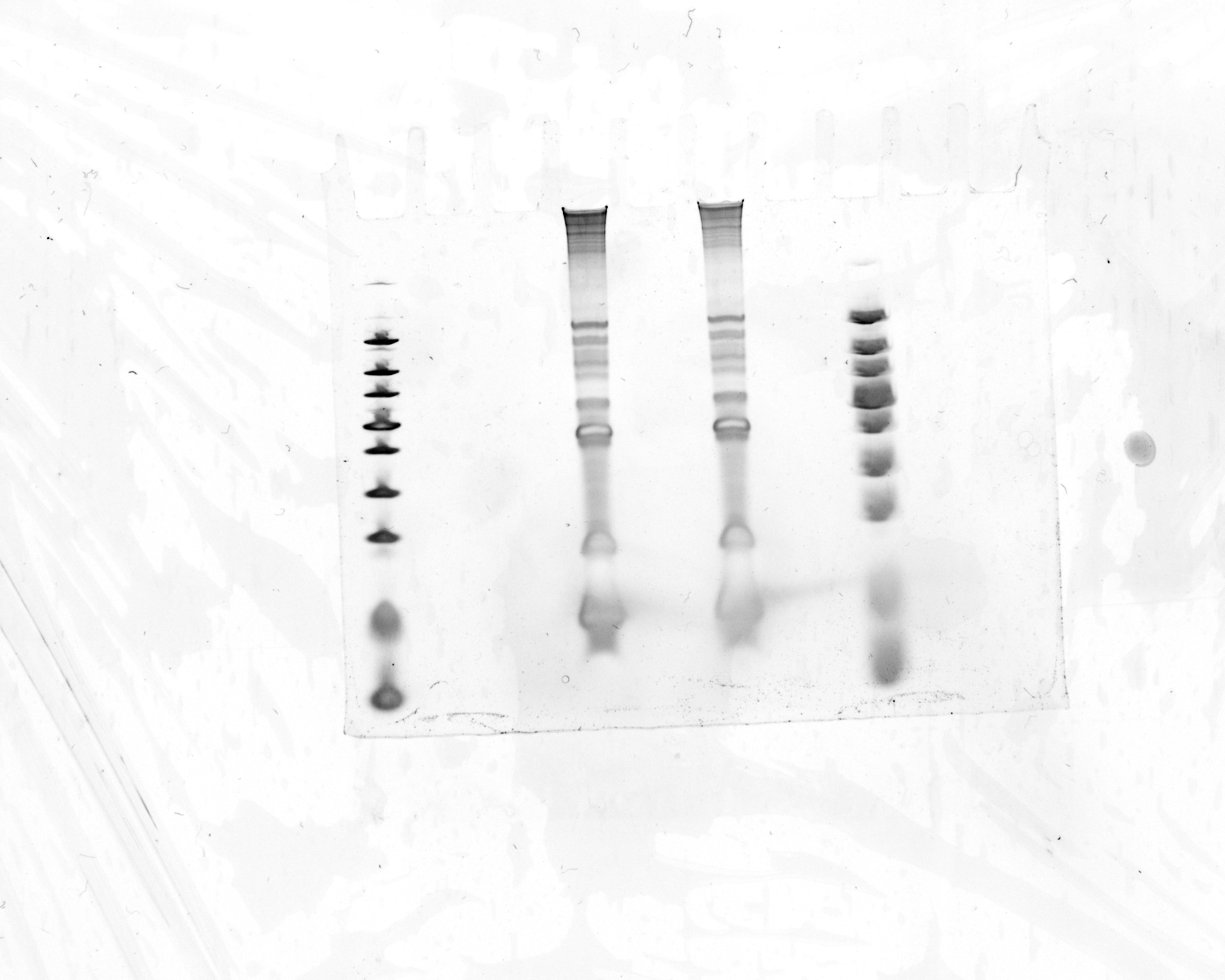

Supplement: Supplementary file 7 — Source Data [file 41467_2020_17879_MOESM7_ESM.zip › Source Data/Raw tif/figure_1c_Hek293_cDNA_pre.tif]

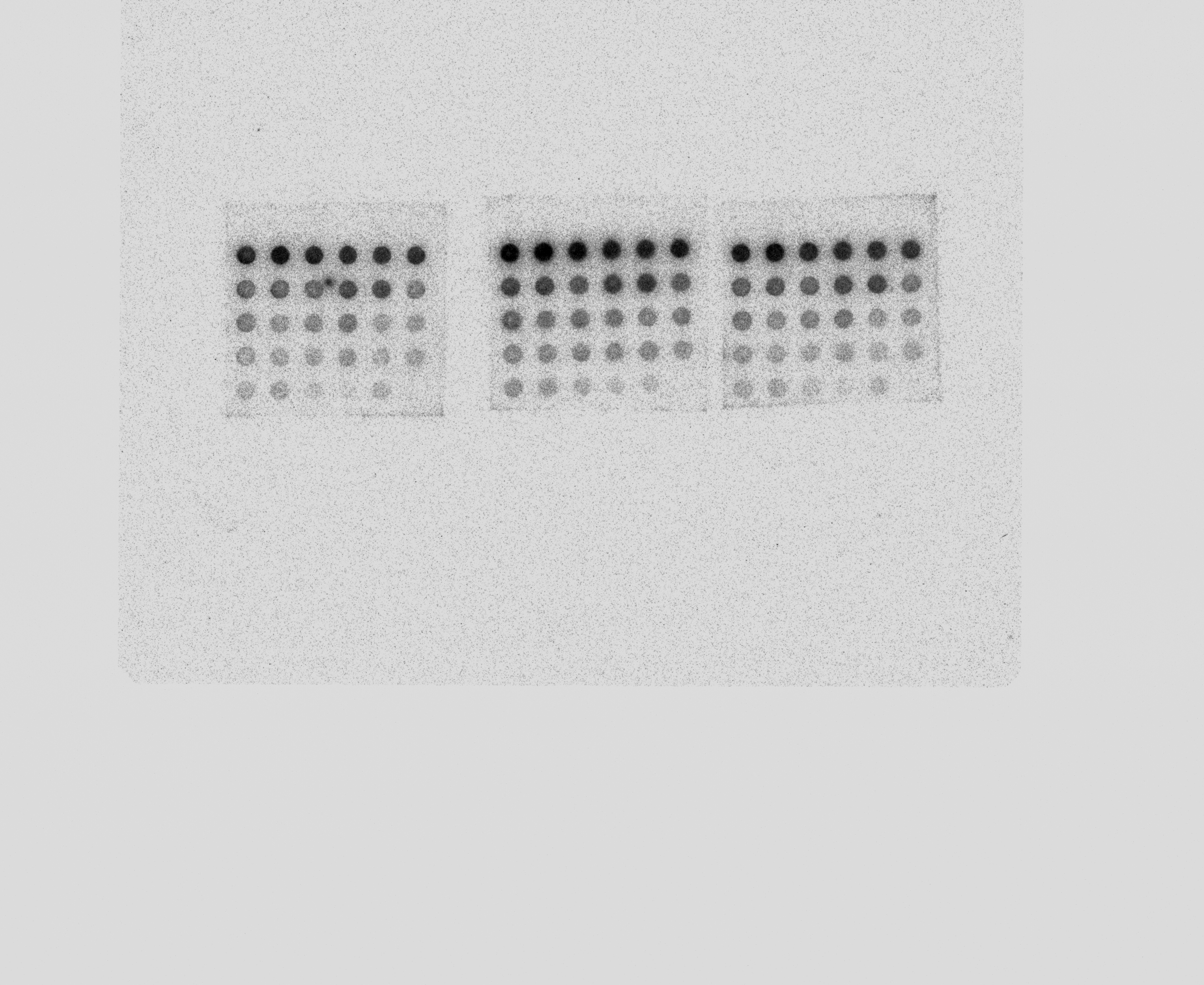

Supplement: Supplementary file 7 — Source Data [file 41467_2020_17879_MOESM7_ESM.zip › Source Data/Raw tif/figure_1d&e_30_array.tif]

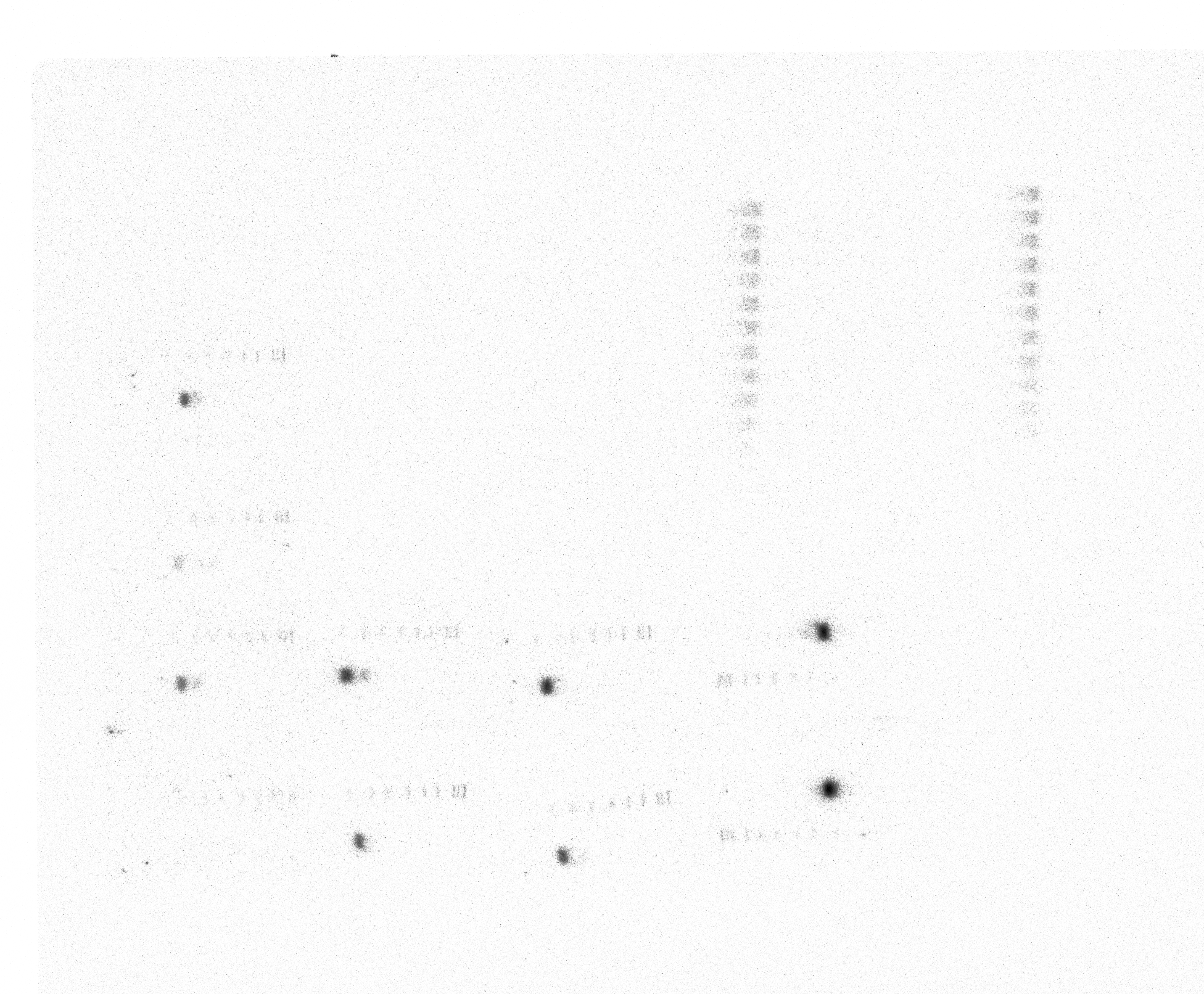

Supplement: Supplementary file 7 — Source Data [file 41467_2020_17879_MOESM7_ESM.zip › Source Data/Raw tif/figure_1f_northern_probes1-10.tif]
